# Epigenetic Drivers of Atrial Fibrillation: Mechanisms, Biomarkers, and Therapeutic Targets

**DOI:** 10.3390/ijms26115253

**Published:** 2025-05-29

**Authors:** Paschalis Karakasis, Panagiotis Theofilis, Nikias Milaras, Panayotis K. Vlachakis, Dimitrios Patoulias, Theodoros Karamitsos, Antonios P. Antoniadis, Nikolaos Fragakis

**Affiliations:** 1 Second Department of Cardiology, Hippokration General Hospital, Aristotle University of Thessaloniki, 54642 Thessaloniki, Greece; aantoniadis@gmail.com (A.P.A.); fragakis.nikos@gmail.com (N.F.); 2 First Cardiology Department, School of Medicine, Hippokration General Hospital, National and Kapodistrian University of Athens, 11527 Athens, Greece; panos.theofilis@hotmail.com (P.T.); nikiasmilaras@gmail.com (N.M.); vlachakispanag@gmail.com (P.K.V.); 3 Second Propedeutic Department of Internal Medicine, Faculty of Medicine, School of Health Sciences Aristotle, University of Thessaloniki, 54642 Thessaloniki, Greece; dipatoulias@gmail.com; 4 First Department of Cardiology, Aristotle University Medical School, AHEPA University General Hospital, 54642 Thessaloniki, Greece; tkaramitsos@auth.gr

**Keywords:** atrial fibrillation, epigenetics, DNA methylation, histone modification, non-coding RNAs, microRNA, chromatin remodeling, RNA methylation, atrial remodeling, SGLT2 inhibitors, epigenetic therapy, biomarkers

## Abstract

Atrial fibrillation (AF) is the most prevalent sustained arrhythmia, associated with significant morbidity, mortality, and healthcare burdens. Despite therapeutic advances, recurrence rates remain high, particularly in persistent AF, underscoring the need for deeper mechanistic insight. Epigenetic regulation—comprising DNA methylation, histone modifications, chromatin remodeling, RNA methylation, and non-coding RNAs—has emerged as a key contributor to the structural, electrical, and inflammatory remodeling underlying AF. These mechanisms operate at the interface of genetic susceptibility and environmental exposure, offering a dynamic framework for understanding disease progression. Systemic stressors such as aging, obesity, diabetes, hypertension, hypoxia, and alcohol have been shown to induce epigenetic reprogramming in atrial tissue, further promoting atrial cardiomyopathy and arrhythmogenesis. Additionally, circulating epigenetic markers, particularly microRNAs, are being investigated for their potential in AF diagnosis, risk stratification, and therapeutic monitoring. Therapeutic strategies targeting epigenetic pathways—ranging from histone deacetylase inhibitors and miRNA-based therapeutics to CRISPR/dCas9-mediated epigenome editing—are under investigation. Additionally, sodium-glucose cotransporter 2 inhibitors may indirectly influence epigenetic programs and miRNA expression relevant to atrial remodeling. While promising, these approaches require further validation in terms of safety, delivery specificity, and long-term efficacy. High-resolution epigenomic mapping and integrative multi-omic approaches may enhance understanding of AF heterogeneity and enable personalized treatment strategies. This review provides an integrated appraisal of epigenetic mechanisms in AF and outlines their emerging diagnostic and therapeutic relevance.

## 1. Introduction

Atrial fibrillation (AF) is the most prevalent sustained cardiac arrhythmia globally, affecting over 37 million individuals and conferring significant risks of stroke, heart failure, cognitive decline, and all-cause mortality [1,2,3,4,5,6,7]. Its incidence is projected to rise steeply in parallel with global aging and the growing burden of cardiometabolic disease [1]. Despite considerable advances in rhythm control strategies, including catheter ablation and antiarrhythmic drug development, AF remains a progressive and often treatment-refractory condition, with recurrence rates exceeding 30–50% within one year following catheter ablation, particularly in its persistent and long-standing forms [8,9,10,11,12,13,14]. The limited efficacy of current therapeutic modalities has underscored the pressing need to re-examine AF pathophysiology beyond its conventional electrophysiological framework [15,16,17,18].

Historically conceptualized as a disorder of electrical instability, AF is increasingly recognized as the clinical manifestation of a broader, structural and molecular substrate known as atrial cardiomyopathy (AtCM) [11,19,20,21,22]. This paradigm shift highlights the complex interplay between atrial fibrosis, inflammation, oxidative stress, and myocyte remodeling, all of which evolve through dynamic gene–environment interactions [23,24,25]. Yet, the molecular underpinnings by which these systemic and local stressors reprogram the atrial substrate remain incompletely defined [19,20]. Emerging evidence now implicates epigenetic mechanisms—heritable but reversible modifications that regulate gene expression without altering the DNA sequence—as critical modulators of AF susceptibility, maintenance, and recurrence [24].

Epigenetic regulation encompasses a spectrum of processes, including DNA methylation, histone post-translational modifications, chromatin remodeling, non-coding RNAs (miRNAs, lncRNAs, circRNAs), and more recently described RNA methylation signatures such as N6-methyladenosine (m6A) [26,27]. These layers of control orchestrate transcriptional landscapes and chromatin architecture that regulate key pathophysiologic events in AF—ranging from ion channel remodeling to fibroblast activation and cellular senescence [28]. Notably, most AF-associated variants identified by genome-wide association studies (GWAS) reside in non-coding genomic regions, implying that transcriptional misregulation—rather than coding mutations—is a predominant mechanism in disease pathogenesis [29,30,31].

Beyond tissue-level alterations, circulating epigenetic signals such as miRNAs and cell-free methylated DNA fragments have emerged as promising biomarkers for AF diagnosis, prognosis, and recurrence prediction following catheter ablation [32,33]. These molecular fingerprints also offer unprecedented potential for patient stratification and precision therapy. Moreover, pharmacologic agents with epigenetic activity—such as histone deacetylase (HDAC) inhibitors, BET bromodomain inhibitors, and RNA-based therapeutics—are gaining momentum as experimental anti-arrhythmic strategies [34,35]. Intriguingly, cardiometabolic drugs like sodium-glucose cotransporter 2 inhibitors (SGLT2i) have been shown to modify miRNA expression profiles and histone acetylation patterns, suggesting an underappreciated capacity to epigenetically modulate the atrial substrate [24].

Growing evidence positions epigenetic remodeling as a central mechanism in AF pathogenesis and a compelling target for precision medicine. This review synthesizes key epigenetic regulators of atrial remodeling, delineates their roles in fibrosis, ion channel dysfunction, and inflammation, evaluates their potential as biomarkers within AtCM, and discusses emerging therapeutic strategies aimed at modulating these pathways.

## 2. Epigenetic Regulation of Structural and Electrical Remodeling in AF

Epigenetics encompasses a spectrum of heritable molecular processes that modulate chromatin architecture and gene expression without altering the underlying DNA sequence [26]. These processes include DNA methylation, histone modifications (such as acetylation, phosphorylation, ubiquitylation, and sumoylation), and higher-order chromatin remodeling [26]. By dynamically regulating chromatin compaction, epigenetic mechanisms influence the accessibility of genomic regions to transcriptional machinery, thereby orchestrating gene activation or repression during key biological events such as transcription, replication, and DNA repair [26]. The epigenome thus plays a fundamental role in both normal cardiovascular development and the pathogenesis of cardiac disease states [27].

While AF has traditionally been viewed as a primarily acquired arrhythmia, the identification of familial AF cases and associated genetic variants has challenged this notion [36]. Contemporary evidence supports a multifactorial model wherein AF arises from a complex interplay between genetic predisposition and environmental or lifestyle-related insults [37]. These influences can converge to disrupt transcriptional homeostasis through epigenetic modifications, ultimately affecting the expression, localization, and function of proteins critical to atrial structure and electrophysiology [38,39].

Genome-wide association studies (GWAS) have established that the majority of AF-associated genetic variants reside within non-coding regions of the genome, implicating regulatory elements rather than protein-coding sequences in disease susceptibility. Notably, key loci such as PITX2, ZFHX3, PRRX1, TBX5, NKX2-5, and HAND2—which influence ion channel expression, gap junction integrity, and cytoskeletal architecture—have been consistently linked to atrial electrophysiological stability and structural homeostasis [29,30,31]. The seminal 2007 GWAS identified two high-risk single nucleotide polymorphisms (SNPs) located in an intergenic region near PITX2, establishing the first robust genetic association with AF in both European and Asian populations [40]. Functional studies have since elucidated the critical role of PITX2 in cardiogenesis, including atrial chamber specification, sinus node development, and left–right asymmetry, with aberrant expression or dosage imbalance of PITX2 contributing to arrhythmogenic remodeling and increased AF susceptibility [41,42].

**Figure 1:**
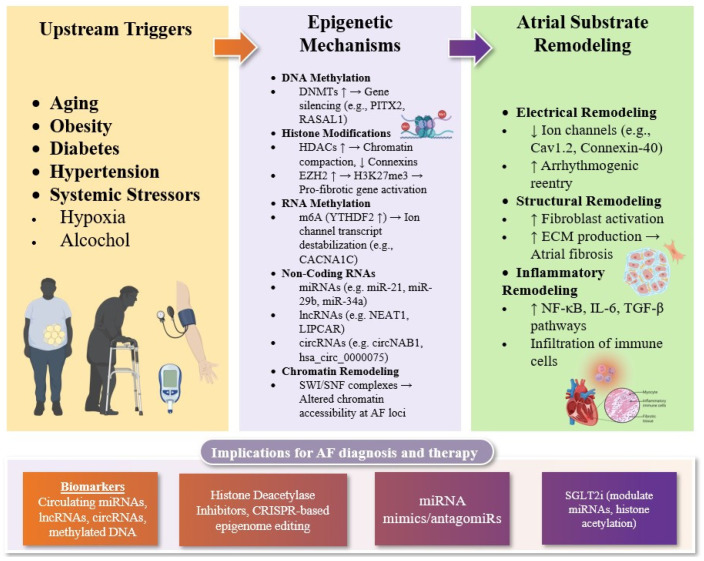
Mechanistic landscape of epigenetic regulation in atrial fibrillation (AF). This conceptual framework illustrates the interplay between systemic stressors (e.g., aging, obesity, diabetes, hypertension, hypoxia, and alcohol) and core epigenetic mechanisms that modulate atrial remodeling in AF. These stressors induce alterations in DNA methylation, histone modifications, RNA methylation, non-coding RNA expression, and chromatin remodeling, which converge to disrupt transcriptional programs governing ion channel expression, fibroblast activation, extracellular matrix turnover, and inflammatory signaling. The resulting electrical, structural, and inflammatory remodeling constitutes the arrhythmogenic substrate that sustains AF. The figure also highlights emerging translational applications, including epigenetic biomarkers (e.g., circulating miRNAs, lncRNAs, and methylated DNA fragments) and therapeutic strategies targeting histone-modifying enzymes, non-coding RNAs, or chromatin states through HDAC inhibitors, RNA-based therapeutics, and CRISPR/dCas9-mediated epigenome editing. Collectively, these mechanisms provide a pathophysiological bridge between genetic susceptibility, environmental exposures, and the atrial phenotype, offering a platform for precision medicine in AF. Abbreviations: AF, atrial fibrillation; AtCM, atrial cardiomyopathy; DNMT, DNA methyltransferase; HDAC, histone deacetylase; HAT, histone acetyltransferase; HMT, histone methyltransferase; EZH2, enhancer of zeste homolog 2; H3K27ac, histone 3 lysine 27 acetylation; H3K27me3, histone 3 lysine 27 trimethylation; m6A, N6-methyladenosine; YTHDF2, YTH domain family protein 2; miRNA, microRNA; lncRNA, long non-coding RNA; circRNA, circular RNA; ECM, extracellular matrix; TGF-β, transforming growth factor beta; MEF2, myocyte enhancer factor-2; NF-κB, nuclear factor kappa-light-chain-enhancer of activated B cells; PITX2, paired-like homeodomain transcription factor 2; CRISPR/dCas9, clustered regularly interspaced short palindromic repeats/deactivated Cas9; SGLT2i, sodium-glucose cotransporter 2 inhibitor.

Building on these findings, subsequent GWAS have revealed additional loci that are functionally interconnected with PITX2 signaling pathways or contribute to atrial substrate vulnerability via transcriptional and epigenetic regulation [43,44]. The cumulative body of evidence now supports the conceptualization of AF as a polygenic and multifactorial disorder, in which inherited genetic risk converges with modifiable environmental and lifestyle-related factors to drive disease initiation and progression. This paradigm underscores the complex and heterogeneous nature of AF pathophysiology, highlighting the importance of integrating genomic, epigenetic, and environmental data in risk stratification and therapeutic targeting (Figure 1).

### 2.1. DNA Methylation and Histone Modifications in AF

DNA methylation is a key epigenetic modification that regulates gene expression at the transcriptional level through the covalent addition of methyl groups to cytosine residues, primarily within CpG dinucleotides [45,46]. This process is catalyzed by DNA methyltransferases (DNMTs), which transfer a methyl group from S-adenosyl-L-methionine to the 5′ carbon of cytosine bases [47,48]. Aberrant DNA methylation patterns have been increasingly recognized as contributors to AF pathogenesis, with differential methylation profiles identified across paroxysmal, persistent, and permanent AF phenotypes [49]. Functionally, DNA methylation can suppress gene expression by preventing transcription factor binding or by recruiting repressive chromatin remodelers. In the context of AF, hypermethylation of genes involved in extracellular matrix regulation and fibroblast activation has been implicated in sustaining atrial fibrosis, a central feature of arrhythmogenic substrate development [50].

Recent genome-wide analyses further underscore the clinical relevance of DNA methylation in AF. In a prospective study of 115 AF patients undergoing catheter ablation, Han et al. [33] identified differential methylation at over 750,000 CpG sites, linking epigenetic alterations to AF subtype, atrial structural remodeling, and recurrence. Among the most significantly hypomethylated genes was DEFB104B (cg20223677), particularly in patients with late recurrence, impaired left atrial (LA) function, and reduced LA appendage flow velocity [33]. These findings suggest that DEFB104B hypomethylation may reflect or contribute to AtCM and predict recurrence post-ablation [33]. Functional enrichment analysis linked DEFB104B to pathways involved in angiogenesis and endothelial cell migration, processes that may contribute to structural remodeling and perpetuation of arrhythmia [33]. These findings suggest that epigenetic regulation of DEFB104B could influence the fibrotic and electrophysiological substrate underlying AF recurrence.

Histone modifications represent another critical axis of epigenetic regulation, influencing chromatin dynamics and gene transcription through post-translational modifications of histone proteins [51]. These modifications, primarily occurring at the N-terminal tails of histones, include acetylation, methylation, phosphorylation, ubiquitylation, and sumoylation [52]. The functional outcome of these modifications depends on their specific nature and location, with acetylation generally associated with transcriptional activation and methylation exerting either activating or repressive effects depending on the residue and methylation state [52]. The epigenetic landscape is dynamically maintained by enzyme families collectively referred to as “writers” (e.g., histone acetyltransferases [HATs], histone methyltransferases [HMTs]), “erasers” (e.g., HDACs, histone demethylases), and “readers” that interpret these marks to modulate downstream gene expression programs [29,53]. In AF, dysregulation of histone-modifying enzymes—particularly HDACs and EZH2—has been linked to structural remodeling and pro-fibrotic transcriptional reprogramming in atrial tissue, positioning histone modifications as central mediators of disease progression.

Dysregulation of histone acetyltransferases (HATs) and HDACs—enzymes that, respectively, add or remove acetyl groups from lysine residues on histone tails—represents one of the most extensively studied epigenetic mechanisms in AF [54]. Aberrant activity of these enzymes has been linked to maladaptive transcriptional reprogramming implicated in both the initiation and progression of AF [34,55]. To date, 18 HDAC isoforms have been identified in humans, several of which have been shown to modulate atrial gene expression networks critical for electrophysiological stability and structural integrity [34]. Notably, epigenome-wide association studies (EWAS) in AF patients have demonstrated increased acetylation of histone H3 at lysine 27 (H3K27ac) in the right atrium, suggesting a mechanistic link between AF-associated genetic variants, chromatin accessibility, and atrial remodeling [56].

Beyond their canonical chromatin-modifying roles, HDACs also regulate the acetylation status of non-histone proteins, thereby influencing key aspects of cardiomyocyte physiology in AF. For example, HDAC-mediated modulation of calcium-handling proteins and transcription factors such as myocyte enhancer factor-2 (MEF2) has been implicated in atrial hypertrophy and fibrosis [57,58,59]. Pharmacological inhibition of HDACs has been shown to reverse atrial structural remodeling, restore calcium homeostasis, and reduce AF susceptibility in preclinical models [57,58,59].

In parallel, histone methylation—another critical epigenetic mark—has also been implicated in the fibrotic remodeling of the atria. In particular, enhancer of zeste homolog 2 (EZH2), the methyltransferase responsible for trimethylation of histone H3 at lysine 27 (H3K27me3), is upregulated in atrial cardiomyocytes and fibroblasts of patients with persistent AF, correlating with profibrotic gene expression and extracellular matrix expansion [28].

Despite these advances, the precise role of DNA methylation and histone modifications in AF pathogenesis remains incompletely defined. It is not yet clear whether these epigenetic changes are causal drivers of disease or secondary consequences of atrial remodeling. Future studies are warranted to clarify the temporal and mechanistic relationships between epigenetic alterations and distinct AF phenotypes, which may ultimately inform targeted therapeutic strategies.

### 2.2. RNA Methylation in AF

RNA methylation constitutes a key epitranscriptomic regulatory mechanism involving covalent chemical modifications on RNA molecules that influence their processing, stability, and function [60,61]. Among these, N^6^-methyladenosine (m^6^A) is the most abundant internal modification in eukaryotic messenger RNA. The deposition of m^6^A marks is catalyzed by a multicomponent methyltransferase complex—referred to as “writers”—comprising enzymes such as METTL3 and METTL14. These modifications are reversible, with demethylases (“erasers”) such as FTO and ALKBH5 capable of removing methyl groups [60,61]. The functional consequences of m^6^A are mediated by a class of RNA-binding proteins known as “readers”, including the YTH domain family proteins (e.g., YTHDF1, YTHDF2, and YTHDF3) [60,61]. Through coordinated action, these factors regulate various aspects of RNA metabolism—such as alternative splicing, nuclear export, transcript stability, and translational efficiency—thereby exerting precise control over gene expression at the post-transcriptional level.

Recent studies have begun to illuminate the role of m^6^A methylation in AF. In a 2024 investigation using a rodent AF model [62], the m^6^A reader protein YTHDF2 was found to be upregulated in atrial cardiomyocytes. Genetic deletion of YTHDF2 significantly reduced AF susceptibility in mice, implicating it in arrhythmia development [62]. Mechanistically, YTHDF2 was shown to bind and destabilize m^6^A-modified transcripts of key ion channel genes—most notably *Cacna1c*, which encodes the L-type calcium channel Cav1.2. Suppression of Cav1.2 expression by YTHDF2 resulted in abbreviated action potential duration and a shortened refractory period, promoting an arrhythmogenic substrate [62]. Conversely, YTHDF2 knockout restored Cav1.2 levels and prolonged refractory periods, exerting a protective, anti-arrhythmic effect [62].

Beyond electrical remodeling, epitranscriptomic profiling of human atrial tissues has revealed distinct m^6^A modification landscapes in AF patients compared to controls [63]. These alterations correlate with changes in immune cell infiltration and pro-inflammatory signaling, suggesting that m^6^A dynamics also contribute to atrial inflammatory remodeling [63]. Collectively, these findings highlight m^6^A methylation as an emerging epigenetic regulator in AF, influencing both ion channel expression and inflammatory pathways, and offering novel therapeutic opportunities.

### 2.3. Chromatin Remodeling in AF

Chromatin remodeling refers to the dynamic reorganization of nucleosome positioning and structure, mediated by ATP-dependent chromatin remodeling complexes such as SWI/SNF, ISWI, CHD, and INO80 [64]. These complexes modulate the accessibility of DNA to transcriptional machinery by repositioning, ejecting, or restructuring nucleosomes, thereby regulating gene expression without altering the underlying histone chemical modifications [29]. Through this mechanism, chromatin remodelers can either facilitate or restrict the exposure of cis-regulatory elements such as enhancers and promoters, influencing transcriptional activity in a context-dependent manner [64].

Although the role of chromatin remodeling in AF is still being elucidated, emerging evidence suggests that altered chromatin accessibility and nucleosome architecture contribute to the epigenetic reprogramming observed in AF [65]. Genome-wide studies have demonstrated that many AF-associated loci identified by GWAS are located in proximity to regions of active chromatin within the atria, implying that changes in chromatin conformation may facilitate the aberrant expression of genes implicated in structural and electrical remodeling [28,56]. Furthermore, chromatin remodeling complexes known to regulate cardiac development and hypertrophy—such as the BRG1-containing SWI/SNF complex—are being investigated for their potential involvement in pathological atrial remodeling [65].

While direct causal links between specific chromatin remodeling factors and human AF remain under active investigation, current evidence supports the notion that chromatin remodeling operates in concert with DNA methylation and histone modifications to orchestrate the gene expression programs driving fibrosis, hypertrophy, and pro-arrhythmic substrate formation [66,67]. Ongoing epigenomic profiling efforts, including assays of chromatin accessibility (e.g., ATAC-seq), are expected to further clarify the contribution of nucleosome remodeling to AF pathophysiology.

### 2.4. Non-Coding RNAs in AF

#### 2.4.1. MicroRNAs

MicroRNAs (miRNAs) are ~22-nucleotide, non-coding RNAs transcribed by RNA polymerase II that regulate gene expression post-transcriptionally by binding to complementary sites in the 3′UTR of target mRNAs [68]. They play essential roles in cellular processes such as growth, differentiation, and metabolism, and are implicated in a range of diseases, including cardiovascular disorders [69,70]. In heart failure and AF, altered miRNA expression contributes to electrical and structural remodeling [32]. MiRNAs are both targets and regulators of epigenetic mechanisms—they can be modulated by DNA methylation and histone modifications, and in turn, repress epigenetic enzymes or recruit chromatin-modifying proteins to gene promoters.

miRNAs are highly expressed in cardiac tissue and serve as critical regulators of electrical stability. Dysregulation of specific miRNAs has been closely linked to pathological electrical remodeling in AF [71] (Table 1). Among them, miR-1 plays a pivotal role; its overexpression enhances delayed rectifier potassium current by downregulating KCNE1 and KCNB2, contributing to the shortening of the atrial effective refractory period [72]. Paradoxically, reduced miR-1 expression has been observed in elderly individuals and patients with persistent AF, indicating a context-dependent modulation of its electrophysiological effects [73,74]. In addition, miR-1 has been shown to reduce intracellular calcium levels, further influencing cardiac excitability [75].

Other miRNAs also modulate calcium handling and ion channel function. Upregulation of miR-21 in atrial cardiomyocytes suppresses expression of voltage-gated calcium channel subunits CACNA1C and CACNB2, thereby diminishing calcium influx [76]. Similarly, miR-328 promotes AF by downregulating CACNA1C and CACNB1, leading to reduced L-type calcium current and shortened action potential duration [77]. Altered calcium signaling also induces miR-499 expression, which downregulates calcium-activated potassium channels, further contributing to electrical remodeling [78].

Dysregulated sarcoplasmic reticulum calcium release via RYR2 is another arrhythmogenic mechanism in AF. Overexpression of miR-106b-25 and miR-208b in AF patients has been associated with increased RYR2 expression and reduced SERCA2 levels, respectively, disrupting calcium cycling and promoting pro-arrhythmic remodeling [79,80,91]. Collectively, these findings highlight the central role of miRNAs in regulating ion channel expression and calcium dynamics, underscoring their contribution to the electrophysiological substrate of AF.

AF is characterized by progressive structural remodeling of the myocardium, including extensive fibrosis [23]. In this context, numerous miRNAs have emerged as key modulators of fibrotic remodeling by regulating genes involved in extracellular matrix (ECM) synthesis and turnover [92]. Among these, miR-21 is notably upregulated in cardiac fibroblasts during AF and has been implicated in the promotion of atrial fibrosis by repressing signaling pathways that ordinarily limit ECM accumulation [81,82]. This dysregulation enhances fibroblast activation and collagen deposition, contributing to substrate formation conducive to AF maintenance.

Similarly, miR-29b plays a protective antifibrotic role by targeting a suite of ECM-related genes, including COL1A1, COL3A1, and ELN (elastin) [83]. Downregulation of miR-29b correlates with increased expression of these fibrotic markers and has been associated with AF onset and progression [83]. Notably, circulating levels of miR-29b are significantly reduced in patients with AF, underscoring its potential as a biomarker of structural remodeling [83].

Beyond structural and electrical remodeling, miRNAs are also implicated in autonomic remodeling—a recognized contributor to AF pathogenesis [93]. Alterations in cardiac electrophysiology are closely linked to enhanced vagal tone and aberrant acetylcholine signaling, which can abbreviate atrial action potential duration and promote arrhythmogenicity [94]. In this setting, upregulation of miR-30d and miR-206 has been associated with impaired acetylcholine-mediated potassium current and increased reactive oxygen species (ROS) production through suppression of SOD1 (superoxide dismutase 1). These changes facilitate maladaptive autonomic remodeling and further destabilize atrial electrophysiological balance [95].

Emerging evidence suggests that miR-150 plays a regulatory role in the pathogenesis of AF. Reduced expression of miR-150 has been consistently observed in both platelets and serum of AF patients, implicating its involvement in atrial remodeling, inflammatory signaling, and platelet activation pathways [84]. A prospective study evaluating patients undergoing catheter ablation for AF reported significantly lower circulating miR-150 levels at baseline compared to healthy controls, with a marked increase following ablation. This dynamic pattern supports a potential cardioprotective function of miR-150 and underscores its capacity to modulate gene networks implicated in structural remodeling of the atria [85].

MiR-483–5p, transcribed from the IGF2 gene locus, has also been identified as a critical mediator of inflammatory signaling in AF. Its upregulation enhances the expression of pro-inflammatory cytokines, including interleukin-6 (IL-6), through activation of the nuclear factor κB (NF-κB) pathway, thereby contributing to the inflammatory milieu associated with AF onset and persistence [86].

In addition to inflammation and remodeling, miRNAs influence vascular homeostasis in the setting of AF. MiR-126, a miRNA highly enriched in cardiac tissue and endothelial cells, is well-established as a key regulator of angiogenesis. In patients with AF, circulating levels of miR-126 have been shown to correlate inversely with disease severity and natriuretic peptide concentrations, suggesting that miR-126 may serve as a biomarker of atrial strain and vascular dysfunction in this context [88].

Single nucleotide polymorphisms (SNPs) represent an additional layer of complexity in the pathogenesis of AF, contributing not only to genetic variation but also to phenotypic heterogeneity [96,97]. miRNAs are known to regulate gene expression under both physiological and pathological conditions, and SNPs occurring within miRNA genes or their biogenesis pathways can significantly influence their expression, processing, or target specificity. Such variations may modulate disease susceptibility, progression, and prognosis across a spectrum of disorders, including neurological diseases, malignancies, metabolic syndromes, and cardiovascular conditions such as AF [96,97].

Recent evidence has identified a functional SNP within the miR-125a gene that contributes to AF pathogenesis by disrupting its interaction with the IL-6 receptor gene. This polymorphism impairs the normal maturation process of miR-125a, leading to dysregulated IL-6 signaling, a pathway strongly implicated in atrial inflammation and remodeling [87]. Similarly, a polymorphism in the precursor sequence of miR-196a2 has been shown to alter the expression of its mature form and weaken its binding affinity to target miRNAs. This disruption enhances susceptibility to AF, likely through downstream effects on gene networks involved in structural and electrophysiological remodeling [98].

While no definitive biomarkers currently exist for the primary diagnosis of AF, circulating miRNAs have emerged as promising candidates due to their stability, sensitivity, and disease-specific expression profiles. Evidence increasingly supports an association between miRNA signatures and AF; however, their expression varies with disease type, severity, and AF subtype or phase. Further validation is required to establish their diagnostic utility. Moreover, early detection of miRNA-related polymorphisms may enhance risk stratification and inform personalized management strategies in AF.

#### 2.4.2. Long Non-Coding RNAs

Long non-coding RNAs (lncRNAs), typically exceeding 200 nucleotides in length, regulate gene expression through a range of mechanisms, including serving as scaffolds for chromatin-modifying complexes, acting as molecular decoys or sponges for RNAs and proteins, or modulating transcription via direct base-pairing with DNA or RNA [99]. Several lncRNAs have been implicated in the pathogenesis of atrial fibrillation (AF), functioning as upstream regulators of structural and electrical remodeling [100] (Table 2).

One well-characterized example is NEAT1, a nuclear-retained lncRNA upregulated in atrial tissue of AF patients. In a murine model, NEAT1 knockdown attenuated angiotensin II–induced atrial fibrosis by releasing miR-320, which in turn repressed the pro-fibrotic transcription factor NPAS2 [101]. This finding highlights the role of NEAT1 as a competing endogenous RNA (ceRNA) that modulates fibrotic signaling pathways [101]. Another study identified the lncRNA TCONS-00106987 as a contributor to electrical remodeling in AF [102]. This lncRNA promotes increased inward-rectifier potassium current (I_K1) by upregulating expression of Kir2.x channel subunits, thereby shortening atrial refractory periods and facilitating the maintenance of reentrant electrical circuits [102,103].

Recent clinical evidence further highlights the translational relevance of lncRNAs in AF staging and substrate characterization. In a prospective study of patients with paroxysmal and persistent AF, UCA1 (urothelial carcinoma–associated 1) expression in right atrial appendage (RAA) tissue was found to significantly correlate with electrophysiological parameters—positively with conduction block and delay, and inversely with conduction velocity [104]. These findings suggest that tissue UCA1 levels reflect the extent of electropathology and may serve as a molecular fingerprint for AF progression. Notably, UCA1 was also elevated in the serum of AF patients, indicating its potential utility as a circulating biomarker [104]. In the same study, two additional lncRNAs— SCOT1-antisense RNA regulating aging in the heart (SARRAH) and Long Intergenic Non-coding RNA Predicting Cardiac Remodeling (LIPCAR)—were found to be downregulated in atrial tissue from AF patients, consistent with prior reports linking them to impaired cardiac contractility, apoptosis, and myocardial remodeling [104]. Paradoxically, their serum levels were elevated in AF, potentially reflecting compensatory release or cell stress–mediated extrusion. Strong intercorrelation among UCA1, SARRAH, and LIPCAR in tissue supports overlapping pathophysiological roles in atrial remodeling [104].

In a study by Wang et al., LIPCAR was shown to regulate atrial fibrosis via the TGF-β/Smad2/3 signaling pathway, a central axis in fibrogenesis [105]. In atrial muscle tissues from AF patients, both LIPCAR and TGF-β1 levels were elevated and positively correlated. Functional assays in human atrial fibroblasts revealed that Ang II stimulation increased LIPCAR expression, along with elevated levels of phosphorylated Smad2/3, α-smooth muscle actin (α-SMA), and collagen types I and III [105]. Overexpression of LIPCAR amplified these effects, promoting fibroblast proliferation and ECM production, while LIPCAR silencing attenuated them [105]. These results position LIPCAR as a pro-fibrotic lncRNA that promotes atrial remodeling via activation of the canonical TGF-β signaling cascade.

Collectively, these findings reinforce the central role of lncRNAs in shaping the fibrotic and electrophysiological substrates of AF. Through diverse mechanisms—including regulation of ion channel expression, modulation of profibrotic signaling, and ceRNA activity—lncRNAs such as LIPCAR contribute to the complex molecular remodeling underlying AF initiation and progression.

#### 2.4.3. Circular RNAs

Circular RNAs (circRNAs) are a distinct class of non-coding RNAs characterized by a covalently closed loop structure, typically generated through back-splicing of exonic sequences [118]. These molecules exert diverse regulatory functions, including acting as miRNA sponges, interacting with RNA-binding proteins, and, in some cases, encoding small peptides [119]. Emerging evidence indicates that circRNAs are abundantly expressed in cardiac tissue and contribute to AF pathophysiology by modulating gene regulatory networks involved in structural and inflammatory remodeling [65,120] (Table 3).

One notable example is circNAB1, a cardiac-enriched circRNA found to be downregulated in AF. Functional studies in murine models have demonstrated that silencing circNAB1 exacerbates atrial fibrosis and inflammation, while its overexpression attenuates collagen deposition and reduces susceptibility to AF [126]. Mechanistically, circNAB1 encodes a regulatory micropeptide, NAB1-356, which interacts with the transcription factor EGR1 to modulate the expression of downstream targets such as RUNX1 and GADD45B, both of which are implicated in pro-fibrotic and pro-inflammatory signaling pathways [126].

In addition to isolated examples, global profiling has revealed widespread alterations in circRNA expression in AF. Comparative transcriptomic studies have identified significant differences in circRNA expression between patients with persistent AF and individuals in sinus rhythm, as well as between rapid atrial pacing (RAP) animal models and controls [125,127]. In one study, 146 differentially expressed circRNAs were detected in RAP-induced AF in dogs, with enrichment analyses pointing toward involvement in cytoskeletal architecture and ion channel regulation [128,129]. Further integrated analyses suggest that circRNA–miRNA–mRNA networks participate in AF pathogenesis through complex regulatory interactions, including competing endogenous RNA (ceRNA) activity [130]. CircRNAs exhibit high stability due to their closed-loop structure, rendering them resistant to exonucleolytic degradation. This stability enhances their potential as regulators and biomarkers in disease contexts. Their well-established ability to sequester miRNAs may contribute to the downregulation of key miRNAs implicated in the transition from paroxysmal to permanent AF [123,131].

Moreover, emerging data implicate dysregulated circRNAs in AF-associated inflammation. In transcriptomic analyses comparing AF patients with healthy controls, over 14,000 differentially expressed circRNAs were identified. Among these, hsa_circ_0000075 and hsa_circ_0082096 were specifically enriched in the transforming growth factor-beta (TGF-β) signaling pathway—a pathway critically involved in atrial fibrosis and AF maintenance [132,133]. Additional enrichment analyses revealed circRNAs associated with cytokine–cytokine receptor interaction, further supporting their role in orchestrating inflammatory responses in AF [133].

Collectively, these findings highlight the complex regulatory landscape of circRNAs in AF, implicating them in structural remodeling, electrophysiological alterations, inflammatory signaling, and cross-talk with other non-coding RNAs. Their stability, functional versatility, and disease-specific expression profiles make them attractive candidates for future biomarker development and therapeutic targeting in AF.

## 3. Modifiers of Atrial Epigenetics: The Role of Systemic Stressors

Emerging evidence indicates that systemic stressors can remodel the atrial epigenome, thereby promoting the atrial cardiomyopathy that underlies atrial fibrillation AF [134,135]. Factors such as advanced age, obesity, diabetes mellitus (DM), hypertension, and environmental exposures trigger oxidative stress and inflammatory signaling, which in turn alter DNA methylation patterns, histone marks, and non-coding RNA expression in atrial cells [136,137]. These epigenetic modifications can silence cardioprotective genes or activate pro-fibrotic and pro-arrhythmic pathways, creating a substrate for atrial structural remodeling and electrical instability.

### 3.1. Aging

Aging is a well-established risk factor for AF and is associated with characteristic epigenetic alterations, including global DNA hypomethylation and locus-specific promoter hypermethylation [138,139]. In both aged human atrial tissue and animal models, these changes have been observed to dysregulate gene expression by derepressing transposable elements while silencing protective gene promoters [140]. Aging-related oxidative stress plays a pivotal role in this process, as increased reactive oxygen species (ROS) disrupt the activity of epigenetic modifiers such as DNA methyltransferases and histone-modifying enzymes [136,137], potentially activating latent pro-fibrotic gene programs. A paradigmatic example involves the transcription factor PITX2, a key determinant of left atrial identity and electrical stability. Studies have shown that in both AF patients and aging hypertensive rats, the PITX2 promoter undergoes aberrant hypermethylation, resulting in its transcriptional repression [141]. This epigenetic silencing is associated with enhanced atrial fibrosis and increased susceptibility to electrical remodeling. Importantly, these age-related epigenetic changes may be at least partially reversible [142]. In experimental models, administration of the DNA methylation inhibitor 5-aza-2′-deoxycytidine successfully restored PITX2 expression and reduced atrial fibrosis [143], suggesting that even established epigenetic modifications in the aging atria may be amenable to therapeutic intervention.

### 3.2. Obesity

Obesity imposes systemic metabolic stress that drives atrial epigenetic remodeling [144]. Adipose tissue produces excess angiotensin II and inflammatory cytokines (e.g., IL-6, TNF-α), activating downstream pathways (ROS generation, NF-κB, TGF-β/Smad) that alter gene regulation [144]. In diet-induced obesity models, researchers have observed widespread changes in cardiac microRNA profiles, as well as chromatin modifications [145]. For instance, high-fat diet feeding in rodents increases vulnerability to AF by slowing atrial conduction through miR-27b–mediated downregulation of connexin-40, a key gap junction protein [145]. This epigenetic effect (elevated miR-27b) impairs electrical coupling and promotes reentrant arrhythmias, independent of acute inflammation [145]. Beyond this, Western diet models show dozens of myocardial miRNAs dysregulated (up to 88 miRNAs altered), enriching pathways like insulin signaling and fibrosis [146]. Notably, pro-fibrotic microRNAs such as miR-21 and miR-29 are upregulated in obese hearts, which can stimulate fibroblast collagen synthesis. Concurrently, obesity-related ROS may inhibit cardiac sirtuins (class III histone deacetylases), leading to hyperacetylation of pro-fibrotic gene promoters [146]. Encouragingly, weight loss has been shown to partially reverse these changes: clinical studies report that intentional weight reduction significantly lowers AF burden and recurrence [147]. This suggests that obesity-driven epigenetic alterations (fibrogenic miRNAs, chromatin marks) are dynamic and can be favorably remodeled by risk factor management, highlighting a route to precision lifestyle therapy in AF.

### 3.3. Diabetes Mellitus

In diabetes, chronic hyperglycemia and insulin resistance provoke a cascade of epigenomic changes that promote atrial cardiomyopathy [148,149]. High glucose levels drive excessive ROS production and advanced glycation end-products, which activate inflammatory and profibrotic signaling (e.g., TGF-β1 and angiotensin II) [150]. These signals converge on the nucleus to alter DNA methylation and histone acetylation. One well-characterized mechanism is the downregulation of miR-133a (a cardiomyocyte-enriched microRNA) in diabetic hearts, which releases its inhibitory hold on DNA methyltransferases [151]. In a murine DM model, loss of miR-133a led to upregulation of DNMT1 and DNMT3b, increasing methylation of target gene promoters [151]. Acute hyperglycemia was shown to elevate DNMT1 (maintenance methylation activity) in atrial myocytes, an effect blunted by restoring miR-133a [151]. The result of this DNMT activation is aberrant CpG methylation and gene silencing—for example, diabetes can induce hypermethylation of anti-fibrotic genes such as RASAL1, a Ras-GTPase activating protein, thereby sustaining fibroblast activation [152]. Diabetes also skews non-coding RNA expression: hyperglycemia drives up miR-21-3p, a microRNA implicated in diabetic atrial fibrosis [153]. Elevated miR-21 (and its 3p strand) in the atrium suppresses PTEN and other negative regulators of fibrosis, facilitating TGF-β/Smad3-mediated collagen deposition [153]. Together, these changes contribute to atrial fibrosis, hypertrophy, and conduction abnormalities in diabetic patients. On a positive note, many diabetes-induced epigenetic changes are potentially reversible. Strict glycemic control and drugs that reduce oxidative stress may indirectly normalize some epigenetic marks. Early experimental therapies are also promising: forced overexpression of miR-133a can reverse DNMT upregulation [151], and inhibitors of TGF-β or angiotensin signaling can prevent hypermethylation of fibrosis suppressors. Such findings raise the prospect of tailoring AF treatments to a patient’s metabolic–epigenetic profile.

### 3.4. Hypertension

Hypertension contributes to the pathogenesis of AF primarily through neurohormonal activation—particularly via the renin–angiotensin–aldosterone system (RAAS)—and mechanical pressure overload, both of which promote epigenetic remodeling within the atrial myocardium [154,155]. Angiotensin II (Ang II), a key effector of RAAS, has been identified as a central mediator linking elevated blood pressure to DNA methylation changes [156]. In atrial cardiomyocytes and fibroblasts, Ang II stimulation through angiotensin type 1 (AT_1_) receptors enhances the expression and activity of DNA methyltransferase 1 (DNMT1), leading to hypermethylation of critical gene promoters, such as Pitx2c. In an Ang II–infused rat model, this promoter hypermethylation was associated with suppressed Pitx2c expression and the development of atrial fibrosis [157]. Notably, these epigenetic alterations were mitigated by pharmacologic intervention: administration of either Losartan (an AT_1_ receptor antagonist) or 5-aza-2′-deoxycytidine (a DNA methylation inhibitor) prevented Pitx2c silencing and attenuated fibrotic remodeling, demonstrating that RAAS-induced epigenetic modifications are therapeutically reversible [157].

Beyond DNA methylation, Ang II excess activates additional pathogenic pathways—including TGF-β/Smad signaling and oxidative stress—that reshape the chromatin landscape. Chronic Ang II exposure recruits chromatin-modifying enzymes that deposit repressive epigenetic marks at essential transcription factor loci [158]. For example, TBX5 and GATA4, which are key regulators of atrial electrophysiological homeostasis, become transcriptionally silenced under hypertensive conditions due to increased DNA and histone methylation at their promoters [158]. This chromatin compaction results in the downstream suppression of ion channel and gap junction gene expression, contributing to slowed atrial conduction and heightened arrhythmic vulnerability. Concurrently, hypertension-induced oxidative stress enhances the expression of profibrotic microRNAs, such as miR-21 [159,160], which further amplify fibroblast activation and extracellular matrix deposition [161,162].

Importantly, these maladaptive epigenetic responses to hypertension are modifiable. Blood pressure control and RAAS inhibition not only reduce hemodynamic burden but may also reverse or prevent the establishment of a pro-arrhythmic epigenetic milieu. Thus, the hypertensive atrial substrate exemplifies how upstream mediators—such as Ang II, ROS, and TGF-β—can drive epigenomic dysregulation, and how their targeted modulation offers a rational and potentially transformative strategy in the prevention and treatment of AF.

### 3.5. Hypoxia

Chronic hypoxia constitutes a significant environmental stressor that may epigenetically predispose the atrial myocardium to arrhythmogenesis [163,164]. Clinical conditions such as obstructive sleep apnea—characterized by intermittent nocturnal hypoxemia—and chronic pulmonary diseases activate the hypoxia-inducible factor-1 alpha (HIF-1α) signaling pathway within cardiac tissues [165]. HIF-1α, in concert with profibrotic mediators such as TGF-β, orchestrates transcriptional reprogramming that promotes structural remodeling [165]. One key consequence of this hypoxic milieu is the epigenetic silencing of antifibrotic genes through aberrant DNA methylation [166]. In endothelial cells, co-activation of hypoxia and TGF-β signaling has been shown to induce hypermethylation of the RASAL1 promoter, leading to transcriptional repression and sustained Ras signaling—a process that drives endothelial-to-mesenchymal transition and fibrotic transformation [167]. A parallel mechanism is likely operative in atrial fibroblasts, where RASAL1 silencing contributes to extracellular matrix accumulation and atrial fibrosis [168].

Moreover, hypoxia impairs the function of oxygen-dependent histone demethylases, resulting in the accumulation of repressive histone methylation marks at key regulatory loci [169]. These modifications can enforce a stable pro-fibrotic transcriptional profile, exemplified by persistent activation of COL1A2 and downregulation of genes encoding connexins and calcium-handling proteins—hallmarks of impaired conduction and atrial contractile dysfunction [169]. Such changes establish a permissive substrate for AF maintenance.

Importantly, hypoxia-induced epigenetic remodeling may be modifiable. In patients with obstructive sleep apnea, therapeutic use of continuous positive airway pressure (CPAP) has been associated with a reduction in AF burden, presumably by mitigating hypoxia-driven molecular reprogramming [170,171]. Although direct epigenetic therapies targeting hypoxia are not yet clinically available, experimental agents such as HIF-1α inhibitors and prolyl hydroxylase domain (PHD) stabilizers are under investigation for their potential to reverse hypoxia-induced transcriptional and epigenetic dysregulation.

### 3.6. Alcohol

Excessive alcohol consumption—commonly referred to as “holiday heart” syndrome—has long been recognized as an acute trigger for AF, while chronic alcohol abuse exerts more insidious effects on the atrial substrate, in part through epigenetic mechanisms [172]. The metabolism of ethanol yields acetaldehyde and generates reactive oxygen species (ROS), both of which can inflict direct genotoxic damage and disrupt redox homeostasis by depleting cellular NAD^+^ reserves. These redox perturbations critically impair the function of sirtuins, particularly SIRT1, a NAD^+^-dependent class III histone deacetylase involved in chromatin regulation and suppression of fibrotic and inflammatory gene expression [173].

Recent experimental data highlight a key epigenetic axis involving miR-34a and SIRT1 [174]. In rodent models, chronic ethanol exposure induces hypomethylation of the miR-34a promoter, leading to its transcriptional upregulation. miR-34a subsequently downregulates SIRT1, resulting in derepression of pro-fibrotic and pro-apoptotic gene networks [175]. This ethanol-driven miR-34a–SIRT1 circuit facilitates histone hyperacetylation and chromatin decompaction at promoters of inflammatory and matrix-remodeling genes [176], thereby fostering a molecular milieu conducive to atrial dilation, interstitial fibrosis, and electrophysiological heterogeneity—hallmarks of an arrhythmogenic substrate.

Importantly, the structural and epigenetic consequences of chronic alcohol exposure are not irreversible. Clinical studies have demonstrated that sustained alcohol abstinence is associated with regression of atrial enlargement and a significant reduction in AF recurrence [177]. These observations suggest that ethanol-induced epigenetic remodeling is dynamic and potentially amenable to reversal. From a precision medicine perspective, targeting the molecular aftermath of alcohol exposure—such as restoring SIRT1 activity through pharmacologic activators (e.g., resveratrol)—may represent a promising adjunctive strategy in alcohol-associated AF. Moreover, identification of alcohol-specific epigenetic signatures, including elevated circulating miR-34a or diminished SIRT1 expression, may facilitate individualized risk stratification and inform tailored management plans that emphasize behavioral modification and potentially epigenetic intervention.

## 4. Emerging Epigenetic Therapies for AF

### 4.1. Histone Deacetylase Inhibitors

Histone acetylation is a reversible chromatin modification that regulates DNA accessibility and transcriptional activity. In the setting of AF, aberrant activity of HDACs has been implicated in the repression of protective genes and the activation of profibrotic and pro-inflammatory pathways [178]. Preclinical studies using broad-spectrum HDAC inhibitors, such as trichostatin A and valproic acid, have demonstrated suppression of atrial fibrosis, attenuation of myofibroblast differentiation, and reduced AF inducibility in rodent and porcine models [179,180]. Notably, HDAC inhibition has been shown to downregulate TGF-β/Smad signaling [181], a central driver of atrial fibrogenesis, and to restore the expression of key regulators such as MEF2 and connexins [182].

Despite these promising findings, translation into clinical practice is hampered by concerns over off-target effects and lack of cardiac specificity. HDACs are ubiquitously expressed and play essential roles in numerous tissues, raising the risk of systemic toxicity. Consequently, current efforts are focused on the development of isoform-selective HDAC inhibitors—particularly targeting HDAC6 or HDAC9 [183,184,185,186], which exhibit enriched expression in cardiac fibroblasts—or on advanced delivery systems that enable atrial-specific targeting.

### 4.2. miRNA-Based Therapies

miRNAs represent a crucial layer of post-transcriptional regulation, and their dysregulation is intimately linked to AF-related remodeling processes. Several miRNAs—including miR-21, miR-29b, miR-34a, and miR-328—have been shown to modulate fibrosis, ion channel expression, calcium handling, and inflammatory cascades in atrial tissue [90,187,188]. Therapeutic strategies aimed at modulating miRNA activity fall into two categories: antagomiRs that inhibit deleterious miRNAs, and miRNA mimics that replenish the function of protective ones [189].

In animal models of AF, silencing of miR-21 via antagomiRs reduced atrial fibroblast activation and collagen synthesis [190], while inhibition of miR-34a restored SIRT1 expression and mitigated histone hyperacetylation [191]. Conversely, administration of miR-29b mimics [192]—known to suppress extracellular matrix genes—has demonstrated anti-fibrotic effects in cardiac tissue [193].

However, the clinical translation of miRNA-based therapies is challenged by issues of tissue-specific delivery, off-target interactions, and immune activation. Advances in nanoparticle encapsulation, viral vectors, and exosome-mediated transport are currently being explored to overcome these limitations [194,195,196]. A small number of miRNA-targeting therapies have entered early-phase human trials in non-cardiac contexts, offering a proof of concept for broader application in AF.

### 4.3. CRISPR-Based Epigenome Editing

The advent of CRISPR-dCas9–based systems has ushered in a new era of precise epigenome editing, allowing targeted modification of DNA methylation or histone marks without altering the underlying genetic code [197,198]. These platforms employ catalytically inactive Cas9 (dCas9) fused to functional domains—such as DNA methyltransferases (e.g., DNMT3A), demethylases (e.g., TET1), or histone acetyltransferases (e.g., p300)—to selectively reprogram chromatin states at specific loci [199,200,201].

In the context of AF, one conceptual application involves demethylating the hypermethylated PITX2c promoter in atrial cardiomyocytes, thereby restoring its expression and maintaining left atrial identity and electrical stability [202,203,204]. Similarly, CRISPR-dCas9-p300 could be used to increase histone acetylation at silenced antifibrotic genes, promoting transcriptional reactivation.

While the specificity and permanence of CRISPR-based epigenetic modulation are highly appealing, several challenges must be addressed prior to clinical application [205]. These include ensuring efficient cardiac delivery, minimizing off-target effects, mitigating immunogenic responses, and resolving regulatory and ethical concerns [205]. Nonetheless, this platform holds immense promise for the long-term reprogramming of arrhythmogenic substrates in AF, particularly in patients with persistent disease or those refractory to current therapies.

### 4.4. Sodium-Glucose Cotransporter 2 Inhibitors

Recent advances in cardiometabolic therapeutics have unveiled the sodium-glucose cotransporter 2 inhibitors (SGLT2i) as agents with pleiotropic effects extending far beyond glycemic control [206,207,208,209,210,211,212,213]. Among the most compelling and novel mechanisms under investigation is their capacity to influence epigenetic and post-transcriptional regulatory pathways—particularly through modulation of miRNA expression profiles and histone modifications. These intersecting molecular effects may contribute to the observed cardiovascular and renal benefits of SGLT2i, including their emerging potential to modify atrial remodeling processes relevant to AF pathogenesis [210].

One of the most innovative hypotheses to explain the cardioprotective properties of SGLT2i centers on their ability to reshape the circulating and tissue-specific miRNA landscape. In patients with heart failure and concomitant diabetes, distinct alterations in miRNA expression have been reported, including downregulation of miR-126, miR-342–3p, and miR-638, and upregulation of pro-inflammatory miR-21 and miR-92, compared to healthy individuals [214]. Interestingly, treatment with empagliflozin has been associated with reductions in circulating miR-21 and miR-92 levels, while such changes are not observed with other glucose-lowering therapies such as metformin or insulin, highlighting a potential SGLT2i-specific effect on miRNA signaling [215].

Furthermore, dapagliflozin has been shown to modulate additional miRNAs relevant to cardiac and renal physiology. It upregulates miR-30e-5p, a regulator that inhibits myocardial autophagy and is typically downregulated in failing hearts [216,217]. Concurrently, dapagliflozin lowers miR-199a-3p, a miRNA whose suppression is thought to facilitate mitochondrial fatty acid oxidation and improve cardiac energetics [218]. These shifts in miRNA expression may reflect a broader role for SGLT2i in reprogramming maladaptive gene regulatory networks implicated in heart failure. However, whether such mechanisms extend to atrial-specific remodeling in AF remains to be elucidated.

In parallel, emerging evidence suggests that SGLT2i may interact with epigenetic pathways through their metabolic effects on signaling intermediates such as β-hydroxybutyrate (3-HBA) [219,220]. This ketone body, which accumulates in plasma and adipose tissue following SGLT2i treatment, acts as an endogenous inhibitor of HDACs and can induce the expression of antioxidant enzymes such as MnSOD and catalase via increased acetylation of histone H3 lysine residues [219,220]. Notably, dapagliflozin has been shown to promote histone H3 lysine 9 β-hydroxybutyrylation in adipocytes, leading to upregulation of adiponectin—a hormone with anti-inflammatory and metabolic regulatory functions [219]. These findings hint at a broader epigenomic impact of SGLT2i, particularly in epicardial adipose tissue [221], a key contributor to the inflammatory and fibrotic milieu in AF.

Although the direct modulation of epigenetic signatures by SGLT2i in atrial tissue remains speculative, these data suggest a plausible miRNA–epigenetic interaction axis influenced by SGLT2 inhibition. Such effects may be indirect or secondary to systemic improvements in metabolic homeostasis, yet their relevance to atrial structural and electrical remodeling deserves further investigation. High-resolution epigenomic studies that map chromatin states and miRNA profiles before and after SGLT2i exposure will be critical to delineate causal relationships and therapeutic implications. Given the central role of epigenetic dysregulation in cardiometabolic disease, the ability of SGLT2i to modulate these pathways—either directly or through intermediary signals—could represent a paradigm-shifting mechanism underpinning their efficacy in AF and beyond.

Lastly, it should be mentioned that none of the epigenetic therapies discussed—such as HDAC inhibitors, miRNA-targeted approaches, or CRISPR/dCas9-based tools—has, to date, received approval for clinical use specifically in atrial fibrillation. These strategies remain investigational, with limited clinical evidence supporting their application in patient populations where AF is the primary indication.

## 5. Limitations and Future Directions

Despite growing insight into the role of epigenetic regulation in AF, several key limitations and unanswered questions persist. First, the temporal and causal relationships between epigenetic alterations and AF phenotypes remain incompletely understood. It is not yet clear whether these changes are primary drivers of disease or secondary adaptations to atrial remodeling. Longitudinal studies incorporating tissue-specific and circulating epigenetic profiling will be essential to clarify whether such modifications precede or follow structural and electrical remodeling. Second, while numerous epigenetic regulators have been implicated in AF through preclinical studies, their translational relevance to human disease requires further validation. Integrative multi-omic approaches—linking epigenomic, transcriptomic, and proteomic data—may help resolve the mechanistic heterogeneity of AF and identify robust therapeutic targets.

Third, the clinical applicability of epigenetic therapies remains limited by significant translational challenges. Although promising strategies such as histone deacetylase inhibitors, miRNA-targeted therapies, and CRISPR/dCas9-mediated epigenome editing have emerged, key barriers—including tissue-specific delivery, off-target effects, and long-term safety—have not been adequately addressed. Additionally, variability in patient response due to genetic background, environmental exposures, and disease stage further complicates therapeutic predictability. Even for more conventional agents like SGLT2 inhibitors, which may exert indirect epigenetic effects via modulation of histone acetylation and miRNA expression, mechanistic studies specific to atrial remodeling are still lacking.

Importantly, the clinical heterogeneity of AF—ranging from paroxysmal to persistent and long-standing forms—adds an additional layer of complexity to epigenetic research and therapeutic development. Emerging data suggest that distinct epigenetic alterations may underlie specific AF subtypes. For instance, certain circulating miRNAs (e.g., miR-150, miR-21) and DNA methylation patterns appear to vary according to AF phenotype, burden, disease duration, or structural remodeling severity [85]. This variability underscores the need for subtype-specific epigenetic profiling, which could enhance the precision of biomarker discovery and therapeutic targeting. Future studies should incorporate stratification by AF subtype and substrate characteristics, ideally through integrative multi-omic frameworks that combine epigenomic, transcriptomic, and proteomic data. Such approaches may enable the development of tailored epigenetic interventions aligned with the molecular architecture of each patient’s disease.

Finally, while circulating miRNAs and other non-coding RNAs represent attractive candidates for non-invasive biomarker development, their diagnostic and prognostic utility remains to be validated in large, multi-ethnic cohorts with standardized endpoints. Establishing their role in disease stratification, recurrence prediction, and therapy monitoring will be crucial for future clinical translation. Addressing these gaps will be instrumental in harnessing the full potential of epigenetic modulation in precision medicine for AF.

## 6. Conclusions

Epigenetic mechanisms—encompassing DNA methylation, histone modifications, non-coding RNAs, and chromatin remodeling—are increasingly recognized as critical modulators of the structural, electrical, and inflammatory substrates that underlie AF. These molecular pathways not only deepen our mechanistic understanding of AF pathophysiology but also hold considerable promise as sources of novel biomarkers and therapeutic targets.

Yet, despite these advances, important questions remain. The precise temporal relationship between epigenetic alterations and disease progression is incompletely defined, and causality has not been fully established. Future investigations should strive to delineate the epigenetic trajectories that precede or parallel atrial remodeling and to identify patient-specific epigenomic signatures amenable to targeted therapeutic modulation.

As research converges across genetics, systems biology, and translational cardiology, epigenetics emerges as a unifying framework—one that integrates inherited susceptibility with modifiable environmental and metabolic influences to shape the atrial phenotype. Ultimately, leveraging this knowledge may catalyze a shift toward more individualized, mechanism-based strategies for AF prevention, diagnosis, and therapy.

## Figures and Tables

**Table 1:** MicroRNAs in epigenetic regulation of atrial fibrillation.

Author (Year)	miRNA	Target(s)	Experimental Model	Mechanism	Epigenetic Impact	Functional Role in AF Pathogenesis
Jia et al. (2013) [72]	miR-1	KCNE1, KCNB2	Right atrial tachypacing rabbit model	miR-1 overexpression downregulates KCNE1 and KCNB2, increasing IKs current	Post-transcriptional repression via 3′UTR binding (confirmed by luciferase assay)	Shortened atrial effective refractory period (AERP), increased AF inducibility
Li et al. (2015) [74]	miR-1, miR-133	HCN2, HCN4	Human right atrial appendage samples (CABG patients with and without AF)	Age-associated downregulation of miR-1/133 correlates with upregulation of HCN2/4	Post-transcriptional repression of HCN channels; inverse expression pattern supports regulatory role	Increased HCN2/4 expression enhances pacemaker current (If), contributing to AF pathogenesis in aged patients
Girmatsion et al. (2009) [73]	miR-1	Kir2.1 (KCNJ2)	Human left atrial tissue (patients with persistent AF undergoing mitral valve surgery); ex vivo atrial slices	miR-1 downregulation associated with increased Kir2.1 expression and IK1 current; confirmed by pacing-induced downregulation in vitro	Post-transcriptional repression of KCNJ2 by miR-1; expression inversely correlated	Enhanced IK1 stabilizes atrial rotors and promotes AF maintenance
Lu et al. (2015) [75]	miR-1	CACNB2 (β2-subunit of L-type Ca2+ channel)	Plasma from AF patients; neonatal rat cardiomyocytes (in vitro transfection)	miR-1 directly targets CACNB2, confirmed by transfection and protein expression assays	Post-transcriptional inhibition of CACNB2 protein expression	Reduced L-type Ca2+ current (ICaL), leading to shortened action potential duration and increased AF susceptibility
Barana et al. (2014) [76]	miR-21	CACNA1C, CACNB2	Human atrial myocytes (CAF vs. SR); HL-1 cells (transfection); CHO cells (luciferase assay)	miR-21 directly binds to 3′UTRs of CACNA1C and CACNB2, reducing mRNA and protein levels of L-type Ca^2+^ channels	Post-transcriptional repression of calcium channel subunits	Reduced ICa,L density, shortened APD, promoting electrical remodeling and AF maintenance
Lu et al. (2010) [77]	miR-328	CACNA1C, CACNB1	AF patients (rheumatic heart disease); canine AF model (atrial tachypacing); transgenic mice; rat atrial myocytes; HEK293 cells	miR-328 upregulation reduces L-type Ca2+ channel subunit expression; confirmed by luciferase assay and Western blot	Post-transcriptional silencing of CACNA1C and CACNB1 via 3′UTR interaction	Reduced ICaL, shortened APD, increased AF susceptibility and maintenance
Ling et al. (2017) [78]	miR-499	CACNB2	Human atrial tissue (permanent AF vs. SR); HL-1 cells (mouse atrial myocytes); luciferase assay; Argonaute pull-down	miR-499 binds to 3′UTR of CACNB2, repressing protein synthesis without degrading mRNA	Post-transcriptional translational repression of CACNB2 via miRISC recruitment	Reduced CACNB2 levels impair L-type Ca2+ channel function; long-term suppression decreases CACNA1C expression, promoting electrical remodeling in AF
Chiang et al. (2014) [79]	miR-93, miR-106b-25 cluster	RyR2 (ryanodine receptor type-2)	Human atrial tissue (pAF vs. SR); miR-106b-25 knockout mice; HEK293 cells (luciferase assay)	Downregulation of the miR-106b-25 cluster leads to loss of repression of RyR2; miR-93 confirmed to bind RyR2 3′UTR	Post-transcriptional derepression of RyR2 via miRNA silencing loss	Increased RyR2 expression, enhanced SR Ca2+ leak, increased atrial ectopy and AF inducibility
Cañón et al. (2016) [80]	miR-208b	CACNA1C, CACNB2, SERCA2, Sox6	Human atrial myocytes (CAF vs. SR); HL-1 cells (transfection); CHO cells (luciferase assay); ovine AF model	miR-208b overexpression suppresses L-type Ca2+ channel subunits and SERCA2; represses transcriptional regulator Sox6	Post-transcriptional repression via 3′UTR targeting of ion channel genes and Ca2+-handling proteins	Reduced ICa,L and SERCA2 expression; disrupted Ca2+ homeostasis; altered myosin isoform expression; promotes electrical remodeling and AF maintenance
Adam et al. (2012) [81]	miR-21	Sprouty1 (Spry1)	Human left atrial tissue (AF vs. SR); neonatal rat fibroblasts; Rac1-transgenic mice; antagomir-21 treatment	miR-21 expression is upregulated by Rac1 and AngII via CTGF and LOX; miR-21 represses Spry1, promoting profibrotic signaling	Post-transcriptional silencing of Spry1 via 3′UTR targeting by miR-21	Enhanced CTGF and collagen expression, increased fibrosis, structural remodeling contributing to AF maintenance
Cao et al. (2017) [82]	miR-21	CADM1	Human atrial tissue (AF vs. SR); SD rat model (ISO-induced fibrosis); neonatal rat cardiac fibroblasts	miR-21 overexpression represses CADM1, leading to activation of STAT3 signaling in cardiac fibroblasts	Post-transcriptional silencing of CADM1 by miR-21	Increased fibroblast proliferation and STAT3 activation, promoting fibrotic remodeling in AF
Dawson et al. (2013) [83]	miR-29b	COL1A1, COL3A1, FBN1	Canine CHF model (ventricular tachypacing); human plasma and atrial tissue; mouse AAV knockdown model; atrial fibroblasts	miR-29b downregulation leads to derepression of ECM genes; confirmed by sponge-mediated knockdown and overexpression studies	Post-transcriptional repression of fibrosis-related ECM genes	Increased collagen expression, fibrosis, and structural remodeling contributing to AF substrate; miR-29b reduction also observed in AF/CHF patients
Goren et al. (2014) [84]	miR-150	Not specified (platelet-associated expression)	Platelets and serum from HF patients with and without AF; miRNA microarray and RT-PCR	Reduced miR-150 levels in platelets and serum correlate with AF; predictive independent of BNP, troponin, and age	miR-150 downregulation in platelets and serum; may influence inflammatory or fibrotic gene expression indirectly	Associated with AF presence in systolic HF; lower miR-150 may contribute to remodeling or thrombogenicity
McManus et al. (2015) [85]	miR-21, miR-150	Not directly evaluated; associated with atrial remodeling pathways	Plasma from AF vs. non-AF patients (n = 211); right atrial tissue (n = 31); follow-up post-ablation (n = 47)	Plasma levels of miR-21 and miR-150 were significantly lower in AF; both increased after catheter ablation	Circulating downregulation of remodeling-associated miRNAs in AF; reversed post-ablation	Suggests miR-21 and miR-150 as potential biomarkers of AF burden and remodeling activity
Harling et al. (2017) [86]	miR-483-5p	Not specified	CABG patients (POAF vs. SR); right atrial biopsies and serial serum sampling	Upregulation of miR-483-5p in atrial myocardium and pre-operative serum of POAF patients; ROC AUC = 0.78	Putative post-transcriptional modulation related to IGF2 transcription under cardiac stress	Elevated pre-op serum levels predict POAF risk; supports existence of arrhythmogenic substrate
Shen et al. (2018) [87]	miR-125a	IL-6R	Human atrial tissue (ERAF vs. LRAF); HL-1 and HEK293 cells (transfection); luciferase assay	miR-125a expression regulated by rs12976445 SNP; miR-125a binds IL-6R 3′UTR and suppresses its expression	Post-transcriptional silencing of IL-6R by miR-125a; rs12976445 affects miR-125a maturation	Reduced miR-125a in ERAF promotes IL-6R-mediated inflammation and increases AF recurrence risk
Wei et al. (2015) [88]	miR-126	Not directly assessed; associated with vascular/endothelial function	Serum from AF, HF, and AF-HF patients vs. healthy controls	miR-126 downregulated in AF and HF; levels inversely correlated with NT-proBNP and LA diameter, positively with LVEF	Implied regulatory role on endothelial function and heart failure biomarkers	Low miR-126 correlates with worse cardiac function and AF/HF severity; potential circulating biomarker
Ren et al. (2025) [89]	miR-21, miR-27b	TGFβRIII, PTEN, MMP-2 (miR-21); Wnt/β-catenin pathway (miR-27b)	Plasma samples from PeAF patients post-RFCA	miR-21 upregulation promotes atrial fibrosis via TGF-β/Smad and PTEN-AKT pathways; miR-27b may modulate fibrosis via Wnt/β-catenin signaling	Post-transcriptional gene silencing via 3′UTR binding; both miRNAs influence transcriptional regulation of fibrotic genes	Higher levels associated with atrial fibrosis and increased recurrence after ablation
Balan et al. (2025) [90]	miR-328	CACNA1C, CACNB1	Spontaneously hypertensive rat (SHR) model with aging	Upregulation of miR-328 reduces L-type Ca^2+^ current by downregulating CACNA1C and CACNB1, shortening action potential duration and promoting re-entry	Post-transcriptional silencing; correlates with progressive electrical remodeling and arrhythmogenic substrate	Correlated with atrial fibrillation burden and predictive of AF onset in hypertensive rats

Abbreviations: 3′UTR, 3′ untranslated region; AAV, adeno-associated virus; AF, atrial fibrillation; AERP, atrial effective refractory period; APD, action potential duration; BNP, B-type natriuretic peptide; CABG, coronary artery bypass grafting; CADM1, cell adhesion molecule 1; Ca^2+^, calcium; CAF, chronic atrial fibrillation; CHO, Chinese hamster ovary; CHF, congestive heart failure; COL1A1, collagen type I alpha 1 chain; COL3A1, collagen type III alpha 1 chain; ECM, extracellular matrix; ERAF, early recurrence of atrial fibrillation; FBN1, fibrillin-1; HCN, hyperpolarization-activated cyclic nucleotide-gated channel; HF, heart failure; HL-1, immortalized murine atrial cardiomyocytes; ICaL, L-type calcium current; IGF2, insulin-like growth factor 2; IK1, inward rectifier potassium current; IKs, slow delayed rectifier potassium current; IL-6R, interleukin-6 receptor; ISO, isoproterenol; LA, left atrium; LOX, lysyl oxidase; LRAF, late recurrence of atrial fibrillation; LVEF, left ventricular ejection fraction; miRNA, microRNA; NT-proBNP, N-terminal pro–B-type natriuretic peptide; pAF, paroxysmal atrial fibrillation; POAF, postoperative atrial fibrillation; Rac1, Ras-related C3 botulinum toxin substrate 1; ROC, receiver operating characteristic; RyR2, ryanodine receptor 2; SERCA2, sarco/endoplasmic reticulum Ca^2+^ ATPase 2; Spry1, Sprouty RTK signaling antagonist 1; SR, sinus rhythm; STAT3, signal transducer and activator of transcription 3.

**Table 2:** Long non-coding RNAs in epigenetic regulation of atrial fibrillation.

Author (Year)	lncRNA	Target(s)	Experimental Model	Mechanism	Epigenetic Impact	Functional Role in AF Pathogenesis
Dai et al. (2021) [101]	lncRNA NEAT1	NPAS2	Human atrial tissue (AF vs. SR); Ang II-induced murine atrial fibrosis model; cardiac fibroblasts; HEK293T luciferase assay	lncRNA NEAT1 functions as a ceRNA for miR-320, relieving its suppression of NPAS2	Competing endogenous RNA (ceRNA) activity of NEAT1 against miR-320	NEAT1 upregulation promotes NPAS2-mediated fibroblast proliferation, migration, and collagen synthesis; contributes to atrial fibrosis and AF substrate
Du et al. (2020) [102]	lncRNA TCONS-00106987	KCNJ2	Rabbit AF model; primary cardiomyocytes; HEK293T cells; lentiviral transfection	lncRNA TCONS-00106987 acts as a ceRNA, sponging miR-26 to derepress KCNJ2 expression	lncRNA-mediated ceRNA activity relieving miR-26–induced repression of KCNJ2	Upregulation of KCNJ2 enhances IK1 current, shortens AERP, and increases AF inducibility
Li et al. (2017) [103]	lncRNA TCONS_00075467	CACNA1C	Rabbit AF model (RA tachypacing); primary atrial myocytes; HEK293T cells; lentiviral infection	lncRNA TCONS_00075467 sponges miR-328, relieving suppression on CACNA1C; confirmed by luciferase assay and co-infection experiments	ceRNA mechanism: TCONS_00075467 binds and inhibits miR-328, indirectly restoring CACNA1C expression	Downregulation of TCONS_00075467 reduces ICaL, shortens AERP and APD, increases AF inducibility
Ramos et al. (2023) [104]	lncRNAs: UCA1, SARRAH, LIPCAR	Not specified (correlated with electrophysiologic conduction features)	Human right atrial appendage and serum samples (AF vs. sinus rhythm); epicardial mapping during surgery	UCA1 levels inversely correlate with conduction velocity and positively with conduction block/delay; SARRAH and LIPCAR downregulated in RAA	Tissue expression reflects AF-associated electropathology; circulating levels elevated in AF	Potential biomarkers of electropathology severity; UCA1 may serve as a bioelectrical fingerprint for AF staging
Wang et al. (2020) [105]	lncRNA LIPCAR	TGF-β1/Smad2/3 signaling	Human atrial tissue (AF vs. SR); Ang II-treated human atrial fibroblasts; siRNA knockdown and overexpression	LIPCAR upregulation enhances Ang II-induced TGF-β1 expression and Smad2/3 phosphorylation; silencing LIPCAR reverses effects	lncRNA LIPCAR modulates fibrotic signaling via interaction with TGF-β/Smad pathway	Promotes fibroblast proliferation, increases expression of α-SMA, Collagen I/III; contributes to atrial fibrosis in AF
Ruan et al. (2015) [106]	lncRNAs (e.g., uc001eqh.1, ENST00000575612, TCONS_00006371, etc.)	Not specified	Human left atrial appendage tissue from AF vs. non-AF patients; lncRNA microarray; qRT-PCR validation	Differential expression analysis and co-expression networks suggest lncRNA involvement in electrical and structural remodeling	Putative regulatory roles inferred from GO and KEGG enrichment; associated with calcium signaling, RAS, NF-κB pathways	Identified 50 highly conserved lncRNAs potentially modulating pathways linked to fibrosis, APD shortening, and AF maintenance
Xu et al. (2016) [107]	lncRNAs: NONHSAT040387, NONHSAG007503, etc.	Not directly specified; inferred via co-expression with TFs like GATA1, TAF7, and EBF1	Peripheral blood samples from AF patients vs. controls; Agilent human lncRNA microarray; qRT-PCR validation	177 differentially expressed lncRNAs (≥2-fold); co-expression network implicates TFs in regulation of lncRNA expression	LncRNA-associated transcriptional regulation and potential miRNA sponge activity	Aberrant lncRNA expression reflects structural remodeling in AF and identifies potential serum biomarkers
Mei et al. (2018) [108]	lncRNAs: SNORD115-22, BC041938, uc010vaf.1, etc.	Not directly specified; inferred via co-expression with ECM-related and immune genes	Right atrial tissue from RHD patients with permanent AF vs. NSR; lncRNA microarray; RT-qPCR validation	182 differentially expressed lncRNAs (fold change > 1.5); co-expression with mRNAs involved in ECM organization and inflammation	Putative regulatory roles through transcriptional modulation and lncRNA-mRNA networks	Dysregulated lncRNAs may contribute to atrial remodeling, immune responses, and fibrotic processes in AF
Su et al. (2018) [109]	lncRNAs: ENST00000559960, uc004aef.3	Putative: KCNA5 (based on co-expression network)	Human leukocyte samples (PAF vs. control); microarray; qRT-PCR validation; CNC network analysis	ENST00000559960 negatively correlates and uc004aef.3 positively correlates with KCNA5; altered expression associated with electrical remodeling	Transcriptional modulation via co-expression with ion channel genes; mechanism remains putative	Potential regulators of KCNA5 and atrial electrical remodeling; ENST00000559960 upregulated and uc004aef.3 downregulated in PAF
Chen et al. (2016) [110]	lncRNA: AK055347	MSS51, Cyp450, ATP synthase	Human LA-PV vs. LAA tissue (AF patients); H9C2 cardiomyocytes (siRNA knockdown); microarray; Western blot; immunofluorescence	AK055347 knockdown inhibits MSS51, Cyp450, and ATP synthase expression; involved in mitochondrial energy production	Transcriptional regulation of mitochondrial genes and metabolic enzymes	AK055347 promotes cardiomyocyte viability and mitochondrial function; contributes to AF-associated metabolic remodeling
Qian et al. (2019) [111]	lncRNAs GAS5, HOTAIRM1, RP11-296O14.3	Multiple mRNAs via ceRNA lncRNA interactions	Human atrial tissue (AF vs. SR); microarray probe reannotation; bioinformatic network and clustering analysis	Eight lncRNAs (e.g., GAS5, HOTAIRM1, RP11-296O14.3) identified in dysregulated lncRNA-mRNA network using ceRNA theory	lncRNAs regulate mRNA targets by sponging miRNAs; ceRNA pairs shift between disease and normal states	lncRNAs influence fibrosis, calcium handling, and metabolism; strong diagnostic value for AF (AUC 0.99)
Zhao et al. (2020) [112]	lncRNAs: ENST00000477757, ENST00000477227, ENST00000479930, etc.	Putative targets include PDLIM1, NOS3, TTC3, CTSB, KCNA4	Human EAT tissue from patients with persistent AF vs. SR (n = 6 each); RNA-sequencing; CNC network; qRT-PCR validation	Differential expression and co-expression network suggest lncRNA regulation of inflammation, fibrosis, ion channel expression	LncRNA-mediated transcriptional and post-transcriptional regulation in EAT impacting atrial remodeling	Altered adipocytokine signaling, fibrosis, and ion channel remodeling; lncRNAs may mediate EAT–myocardium crosstalk in AF
Lu et al. (2019) [113]	lncRNA: GAS5	ALK5	Human RAA tissues (AF vs. SR); AC16 cardiomyocytes (GAS5 knockdown/overexpression); qRT-PCR; colony assay	GAS5 negatively regulates ALK5; overexpression suppresses, knockdown increases ALK5 expression	Transcriptional regulation of ALK5 by lncRNA GAS5	GAS5 inhibits fibroblast proliferation and attenuates fibrotic remodeling in AF
Sun et al. (2019) [114]	lncRNA: NRON	NFATc3/IL-12	Mouse atrial myocytes; RAW264.7 macrophages; mouse atrial fibroblasts; Ang II treatment; gene transfection	NRON inhibits NFATc3 nuclear transport, suppressing IL-12 transcription; this limits M1 macrophage polarization and fibroblast activation	Transcriptional repression of IL-12 via NFATc3 inhibition; intercellular signaling modulation through conditioned media	Reduced M1 macrophage activation and inflammatory cytokine secretion; attenuated atrial fibroblast-mediated collagen production and fibrosis
Yu et al. (2017) [115]	lncRNAs: TCONS_00076385, TCONS_00194688, TCONS_00024161	Co-expressed with IFI27, IFIT2, IFI6, IDH1, LAMP3, SAMD9L	Human lymphocytes from pmAF patients vs. controls; RNA-seq; qRT-PCR validation; co-expression network	Upregulated lncRNAs co-express with genes involved in IFN signaling, oxidative stress, and autophagy	lncRNA-associated transcriptional modulation of inflammatory and stress-response pathways	May contribute to inflammatory and oxidative stress mechanisms underlying permanent AF pathophysiology
Shen et al. (2018) [116]	lncRNA KCNQ1OT1	CACNA1C	Ang-II-induced AF mouse model; primary atrial cardiomyocytes; HEK293T cells; ChIP and luciferase assays	lncRNA KCNQ1OT1 sponges miR-384, relieving suppression of CACNA1C; YY1 transcription factor upregulates both KCNQ1OT1 and CACNA1C	ceRNA regulation by KCNQ1OT1; YY1-induced transcriptional upregulation	Increased CACNA1C expression enhances ICaL; shortened ERP, increased AF incidence and duration in Ang-II model
Wen et al. (2023) [117]	lncRNA XIST	CADM1	Peripheral blood mononuclear cells from AF vs. healthy controls; microarray; qRT-PCR; ceRNA network; correlation analysis	lncRNA XIST and circRNA_2773 act as ceRNAs that sponge miR-486-5p, relieving suppression of CADM1	Post-transcriptional derepression via competitive endogenous RNA (ceRNA) mechanism	Upregulation of CADM1 promotes PI3K/AKT pathway activation; implicated in AF-related fibrosis and inflammation

Abbreviations: AF, atrial fibrillation; AERP, atrial effective refractory period; AKT, protein kinase B; ALK5, activin receptor-like kinase 5; APD, action potential duration; ceRNA, competing endogenous RNA; CHL1, close homolog of L1; CTSB, cathepsin B; ECM, extracellular matrix; EAT, epicardial adipose tissue; GAS5, growth arrest-specific 5; ICaL, L-type calcium current; IFN, interferon; IL-12, interleukin-12; KCNA4, voltage-gated potassium channel subfamily A member 4; LA-PV, left atrial–pulmonary vein junction; LAA, left atrial appendage; lncRNA, long non-coding RNA; MATR3, matrin 3; miRNA, microRNA; NFATc3, nuclear factor of activated T-cells 3; NOS3, nitric oxide synthase 3; NPAS2, neuronal PAS domain protein 2; PDLIM1, PDZ and LIM domain protein 1; PI3K, phosphoinositide 3-kinase; RAC3, Rac family small GTPase 3; RAA, right atrial appendage; REST, RE1-silencing transcription factor; RNA, ribonucleic acid; Smad, SMAD family transcription factors; Sp1, specificity protein 1; SR, sinus rhythm; STAM, signal transducing adaptor molecule; SUCO, SUN domain-containing ossification factor; TGF-β, transforming growth factor beta; TF, transcription factor; YY1, Yin Yang 1 transcription factor.

**Table 3:** Circular RNAs in epigenetic regulation of atrial fibrillation.

Author (Year)	circRNA	Target(s)	Experimental Model	Mechanism	Epigenetic Impact	Functional Role in AF Pathogenesis
Liu et al. (2023) [121]	hsa_circ_0004214, hsa_circ_0000615, hsa_circ_0003862, hsa_circ_0002202, hsa_circ_0000745, hsa_circ_0008326	miR-103a, miR-107, miR-320d, miR-1468-3p, miR-6736-3p, miR-3194-3p, miR-5580-5p, miR-4518, miR-16-5p	Peripheral blood of PAF patients with and without LRAF (n = 6); circRNA-seq; qRT-PCR validation; circRNA-miRNA interaction network prediction	Differentially expressed circRNAs act as miRNA sponges (ceRNAs), modulating transcription factors and fibrotic/inflammatory pathways	Post-transcriptional modulation of gene expression via circRNA-miRNA interactions	May influence atrial remodeling and fibrosis; potential circulating biomarkers or therapeutic targets for LRAF
Xue et al. (2023) [120]	hsa_circ_0043278, hsa_circ_0000511, hsa_circ_0006220, hsa_circ_0001666	miR-1207, miR-3192, miR-3200, miR-432, miR-187, miR-548, miR-4254, miR-345	Serum samples of CABG patients with and without AF; GSE129409 and GSE97455; qRT-PCR validation; bioinformatics prediction	DEcircRNAs act as miRNA sponges (ceRNAs), regulating mRNAs involved in fibrosis and inflammation; ceRNA network confirmed with miRNA and mRNA databases	Post-transcriptional regulation via circRNA–miRNA–mRNA interactions	Increased circRNA expression linked to AF occurrence and recurrence post-CABG; potential diagnostic biomarkers
Hu et al. (2019) [122]	circRNA_20118, circRNA_17558, circRNA_16688, circRNA_11058, circRNA_11017, circRNA_11109, circRNA_19591, circRNA_19596, circRNA_16175	miR-29b-1-5p, miR-29b-2-5p (and others in circRNA–miRNA co-expression network)	Human left atrial appendage tissue (persistent AF with RHD vs. healthy donor hearts); RNA-seq; qRT-PCR validation; GO and KEGG pathway analyses; circRNA–miRNA network construction	Differentially expressed circRNAs act as miRNA sponges; predicted by miRanda and visualized using Cytoscape	Post-transcriptional repression via circRNA–miRNA interactions	Dysregulated circRNAs may influence fibrosis, inflammation, and cardiomyopathic pathways in AF with RHD; identified as potential regulatory hubs
Costa et al. (2019) [123]	hsa_circ_0025470, hsa_circ_0035132, hsa_circ_0035148, hsa_circ_0057344, hsa_circ_0085900, hsa_circ_0105720, hsa_circ_0112651, hsa_circ_0112664, hsa_circ_0112682	miR-181d-5p, miR-3180-3p, miR-6868-3p, miR-2277-5p (among others)	Left atrial biopsies from patients with permanent AF, paroxysmal AF, and sinus rhythm; RNA-seq; circRNA-miRNA network analysis using miRanda and NAViGaTOR	circRNAs exclusively expressed in permanent AF sponge specific miRNAs, contributing to their downregulation during disease progression	Post-transcriptional repression through circRNA-miRNA interactions	CircRNA-mediated miRNA downregulation contributes to progression from paroxysmal to permanent AF via regulatory ncRNA crosstalk
Ruan et al. (2020) [124]	circRNA_7571, circRNA_4648, circRNA_4631, circRNA_2875	hsa-miR-328 (primary predicted interaction)	Peripheral blood monocytes from AF patients vs. healthy controls (n = 4 per group); circRNA microarray; qRT-PCR validation; circRNA–miRNA network using Cytoscape	Differentially expressed circRNAs act as miRNA sponges; circRNA–miRNA co-expression network identified major hubs	Post-transcriptional regulation of miRNA availability; potential modulation of fibrosis- and inflammation-related targets	Key circRNAs may contribute to AF pathogenesis via miR-328 sequestration; may serve as novel biomarkers or therapeutic targets
Zhu et al. (2020) [125]	circ_255-ITGA7, circ_418-KCNN2, circ_13913-MIB1, circ_44782-LAMA2, circ_81906-RYR2, circ_3136-TNNI3K	miR-302a-3p, miR-302b-3p, miR-302c-3p, miR-302d-3p (RELA); miR-7-5p (CALM2, CALM3)	LAA tissues from VHD patients with vs. without PeAF (n = 10 + 18); RNA-seq; qRT-PCR; Sanger sequencing; circRNA-miRNA-mRNA network prediction	circRNAs function as miRNA sponges; circ_418-KCNN2 regulates RELA via miR-302 family; circ_81906-RYR2 modulates CALM2/3 via miR-7-5p	Post-transcriptional regulation via circRNA-mediated miRNA sequestration	Involvement in calcium handling and inflammatory pathways (e.g., cAMP, Wnt, Rap1); contribute to atrial fibrosis and AF in VHD
Du et al. (2025) [126]	circNAB1	EGR1, Runx1, Gadd45b	Human atrial tissue (AF vs. SR); circNAB1-transgenic and knockout mice; HL-1, AC16, and HCF cell lines; TAC model; LKB1 knockout mice	circNAB1 encodes novel peptide NAB1-356 that interacts with EGR1 and represses transcription of Runx1 and Gadd45b, reducing cytokine expression and fibrosis	Translation of circRNA-derived peptide modulating transcription factor binding and gene expression	Reduces atrial fibrosis and inflammation; decreases AF incidence and duration; potential therapeutic target in AF with pressure overload or genetic predisposition

Abbreviations: AC16, human ventricular cardiomyocyte cell line; AF, atrial fibrillation; CABG, coronary artery bypass grafting; CALM2/3, calmodulin 2/3; cAMP, cyclic adenosine monophosphate; ceRNA, competing endogenous RNA; circRNA, circular RNA; DEcircRNA, differentially expressed circular RNA; EGR1, early growth response 1; Gadd45b, growth arrest and DNA damage-inducible beta; GO, gene ontology; HCF, human cardiac fibroblasts; HL-1, murine atrial cardiomyocyte cell line; KEGG, Kyoto Encyclopedia of Genes and Genomes; LAA, left atrial appendage; LKB1, liver kinase B1; LRAF, late recurrence of atrial fibrillation; miRNA, microRNA; NAViGaTOR, network analysis, visualization, and graphing Toronto software; ncRNA, non-coding RNA; PAF, paroxysmal atrial fibrillation; PeAF, persistent atrial fibrillation; qRT-PCR, quantitative reverse transcription polymerase chain reaction; RHD, rheumatic heart disease; RNA-seq, RNA sequencing; RYR2, ryanodine receptor 2; SR, sinus rhythm; TAC, transverse aortic constriction; TCF25, transcription factor 25; VHD, valvular heart disease; Wnt, Wnt signaling pathway.

## Data Availability

All data generated in this research is included within the article.

## References

[1] Tan S., Zhou J., Veang T., Lin Q., Liu Q. (2025). Global, Regional, and National Burden of Atrial Fibrillation and Atrial Flutter from 1990 to 2021: Sex Differences and Global Burden Projections to 2046—A Systematic Analysis of the Global Burden of Disease Study 2021. Europace.

[2] Kornej J., Börschel C.S., Benjamin E.J., Schnabel R.B. (2020). Epidemiology of Atrial Fibrillation in the 21st Century: Novel Methods and New Insights. Circ. Res..

[3] Linz D., Gawalko M., Betz K., Hendriks J.M., Lip G.Y.H., Vinter N., Guo Y., Johnsen S. (2024). Atrial Fibrillation: Epidemiology, Screening and Digital Health. Lancet Reg. Heal. Eur..

[4] Alonso A., Bengtson L.G.S. (2014). A Rising Tide: The Global Epidemic of Atrial Fibrillation. Circulation.

[5] Linz D., Andrade J.G., Arbelo E., Boriani G., Breithardt G., Camm A.J., Caso V., Nielsen J.C., De Melis M., De Potter T. (2024). Longer and Better Lives for Patients with Atrial Fibrillation: The 9th AFNET/EHRA Consensus Conference. Europace.

[6] Karakasis P., Pamporis K., Siontis K.C., Theofilis P., Samaras A., Patoulias D., Stachteas P., Karagiannidis E., Stavropoulos G., Tzikas A. (2024). Major Clinical Outcomes in Symptomatic vs. Asymptomatic Atrial Fibrillation: A Meta-Analysis. Eur. Heart J..

[7] Pamporis K., Karakasis P., Sagris M., Theofilis P., Milaras N., Pantelidaki A., Mourouzis I., Fragakis N., Vlachos K., Kordalis A. (2025). Prevalence of Asymptomatic Atrial Fibrillation and Risk Factors Associated with Asymptomatic Status: A Systematic Review and Meta-Analysis. Eur. J. Prev. Cardiol..

[8] Packer D.L., Mark D.B., Robb R.A., Monahan K.H., Bahnson T.D., Poole J.E., Noseworthy P.A., Rosenberg Y.D., Jeffries N., Mitchell L.B. (2019). Effect of Catheter Ablation vs Antiarrhythmic Drug Therapy on Mortality, Stroke, Bleeding, and Cardiac Arrest Among Patients With Atrial Fibrillation: The CABANA Randomized Clinical Trial. JAMA.

[9] Verma A., Jiang C., Betts T.R., Chen J., Deisenhofer I., Mantovan R., Macle L., Morillo C.A., Haverkamp W., Weerasooriya R. (2015). Approaches to Catheter Ablation for Persistent Atrial Fibrillation. N. Engl. J. Med..

[10] Karakasis P., Fragakis N., Patoulias D., Theofilis P., Kassimis G., Karamitsos T., El-Tanani M., Rizzo M. (2024). Effects of Glucagon-Like Peptide 1 Receptor Agonists on Atrial Fibrillation Recurrence After Catheter Ablation: A Systematic Review and Meta-Analysis. Adv. Ther..

[11] Goette A., Corradi D., Dobrev D., Aguinaga L., Cabrera J.-A., Chugh S.S., de Groot J.R., Soulat-Dufour L., Fenelon G., Hatem S.N. (2024). Atrial Cardiomyopathy Revisited-Evolution of a Concept: A Clinical Consensus Statement of the European Heart Rhythm Association (EHRA) of the ESC, the Heart Rhythm Society (HRS), the Asian Pacific Heart Rhythm Society (APHRS), and the Latin American Hear. Europace.

[12] Shen M.J., Arora R., Jalife J. (2019). Atrial Myopathy. JACC Basic Transl. Sci..

[13] Boriani G., Gerra L., Mantovani M., Tartaglia E., Mei D.A., Imberti J.F., Vitolo M., Bonini N. (2023). Atrial Cardiomyopathy: An Entity of Emerging Interest in the Clinical Setting. Eur. J. Intern. Med..

[14] Karakasis P., Patoulias D., Popovic D.S., Pamporis K., Theofilis P., Nasoufidou A., Stachteas P., Samaras A., Tzikas A., Giannakoulas G. (2024). Effects of Mineralocorticoid Receptor Antagonists on New-Onset or Recurrent Atrial Fibrillation: A Bayesian and Frequentist Network Meta-Analysis of Randomized Trials. Curr. Probl. Cardiol..

[15] Wilber D.J., Pappone C., Neuzil P., De Paola A., Marchlinski F., Natale A., Macle L., Daoud E.G., Calkins H., Hall B. (2010). Comparison of Antiarrhythmic Drug Therapy and Radiofrequency Catheter Ablation in Patients with Paroxysmal Atrial Fibrillation: A Randomized Controlled Trial. JAMA.

[16] Razzack A.A., Lak H.M., Pothuru S., Rahman S., Hassan S.A., Hussain N., Najeeb H., Reddy K.T., Syeda H., Yasmin F. (2022). Efficacy and Safety of Catheter Ablation vs Antiarrhythmic Drugs as Initial Therapy for Management of Symptomatic Paroxysmal Atrial Fibrillation: A Meta-Analysis. Rev. Cardiovasc. Med..

[17] Sanchez-Somonte P., Kittichamroen N., Gao-Kang J., Azizi Z., Alipour P., Kahykin Y., Pantano A., Verma A. (2024). Incremental Efficacy for Repeat Ablation Procedures for Catheter Ablation of Atrial Fibrillation: 5-Year Follow-Up. JACC Adv..

[18] Ngo L., Lee X.W., Elwashahy M., Arumugam P., Yang I.A., Denman R., Haqqani H., Ranasinghe I. (2023). Freedom from Atrial Arrhythmia and Other Clinical Outcomes at 5 Years and beyond after Catheter Ablation of Atrial Fibrillation: A Systematic Review and Meta-Analysis. Eur. Heart J.-Qual. Care Clin. Outcomes.

[19] Van Gelder I.C., Rienstra M., Bunting K.V., Casado-Arroyo R., Caso V., Crijns H.J.G.M., De Potter T.J.R., Dwight J., Guasti L., Hanke T. (2024). 2024 ESC Guidelines for the Management of Atrial Fibrillation Developed in Collaboration with the European Association for Cardio-Thoracic Surgery (EACTS). Eur. Heart J..

[20] Joglar J.A., Chung M.K., Armbruster A.L., Benjamin E.J., Chyou J.Y., Cronin E.M., Deswal A., Eckhardt L.L., Goldberger Z.D., Gopinathannair R. (2024). 2023 ACC/AHA/ACCP/HRS Guideline for the Diagnosis and Management of Atrial Fibrillation: A Report of the American College of Cardiology/American Heart Association Joint Committee on Clinical Practice Guidelines. Circulation.

[21] Karakasis P., Theofilis P., Vlachakis P.K., Ktenopoulos N., Patoulias D., Antoniadis A.P., Fragakis N. (2025). Atrial Cardiomyopathy in Atrial Fibrillation: Mechanistic Pathways and Emerging Treatment Concepts. J. Clin. Med..

[22] Karakasis P., Vlachakis P.K., Theofilis P., Ktenopoulos N., Patoulias D., Fyntanidou B., Antoniadis A.P., Fragakis N. (2025). Atrial Cardiomyopathy in Atrial Fibrillation: A Multimodal Diagnostic Framework. Diagnostics.

[23] Karakasis P., Theofilis P., Vlachakis P.K., Korantzopoulos P., Patoulias D., Antoniadis A.P., Fragakis N. (2024). Atrial Fibrosis in Atrial Fibrillation: Mechanistic Insights, Diagnostic Challenges, and Emerging Therapeutic Targets. Int. J. Mol. Sci..

[24] Donniacuo M., De Angelis A., Telesca M., Bellocchio G., Riemma M.A., Paolisso P., Scisciola L., Cianflone E., Torella D., Castaldo G. (2023). Atrial Fibrillation: Epigenetic Aspects and Role of Sodium-Glucose Cotransporter 2 Inhibitors. Pharmacol. Res..

[25] Molina C.E., Abu-Taha I.H., Wang Q., Roselló-Díez E., Kamler M., Nattel S., Ravens U., Wehrens X.H.T., Hove-Madsen L., Heijman J. (2018). Profibrotic, Electrical, and Calcium-Handling Remodeling of the Atria in Heart Failure Patients With and Without Atrial Fibrillation. Front. Physiol..

[26] Deans C., Maggert K.A. (2015). What Do You Mean, “Epigenetic”?. Genetics.

[27] Prasher D., Greenway S.C., Singh R.B. (2020). The Impact of Epigenetics on Cardiovascular Disease. Biochem. Cell Biol..

[28] Song S., Zhang R., Mo B., Chen L., Liu L., Yu Y., Cao W., Fang G., Wan Y., Gu Y. (2019). EZH2 as a Novel Therapeutic Target for Atrial Fibrosis and Atrial Fibrillation. J. Mol. Cell. Cardiol..

[29] Gillette T.G., Hill J.A. (2015). Readers, Writers, and Erasers: Chromatin as the Whiteboard of Heart Disease. Circ. Res..

[30] Chiarella A.M., Lu D., Hathaway N.A. (2020). Epigenetic Control of a Local Chromatin Landscape. Int. J. Mol. Sci..

[31] van Ouwerkerk A.F., Bosada F.M., van Duijvenboden K., Hill M.C., Montefiori L.E., Scholman K.T., Liu J., de Vries A.A.F., Boukens B.J., Ellinor P.T. (2019). Identification of Atrial Fibrillation Associated Genes and Functional Non-Coding Variants. Nat. Commun..

[32] Condorelli G., Latronico M.V.G., Cavarretta E. (2014). MicroRNAs in Cardiovascular Diseases: Current Knowledge and the Road Ahead. J. Am. Coll. Cardiol..

[33] Han M.-R., Jeong J.H., Kim Y.G., Yang H.-H., Seo C.-O., Kim Y., Lee H.S., Shim J., Kim Y.-H., Choi J.-I. (2024). Epigenetic Regulation on Left Atrial Function and Disease Recurrence after Catheter Ablation in Atrial Fibrillation. Clin. Epigenetics.

[34] Brundel B.J.J.M., Li J., Zhang D. (2020). Role of HDACs in Cardiac Electropathology: Therapeutic Implications for Atrial Fibrillation. Biochim. Biophys. Acta Mol. Cell Res..

[35] Auguste G., Rouhi L., Matkovich S.J., Coarfa C., Robertson M.J., Czernuszewicz G., Gurha P., Marian A.J. (2020). BET Bromodomain Inhibition Attenuates Cardiac Phenotype in Myocyte-Specific Lamin A/C-Deficient Mice. J. Clin. Investig..

[36] Creemers E.E., Wilde A.A., Pinto Y.M. (2011). Heart Failure: Advances through Genomics. Nat. Rev. Genet..

[37] Sweat M.E., Pu W.T. (2024). Genetic and Molecular Underpinnings of Atrial Fibrillation. NPJ Cardiovasc. Health.

[38] Paludan-Müller C., Svendsen J.H., Olesen M.S. (2016). The Role of Common Genetic Variants in Atrial Fibrillation. J. Electrocardiol..

[39] Lee J., Lee H., El Sherbini A., Baghaie L., Leroy F., Abdel-Qadir H., Szewczuk M.R., El-Diasty M. (2024). Epigenetic MicroRNAs as Prognostic Markers of Postoperative Atrial Fibrillation: A Systematic Review. Curr. Probl. Cardiol..

[40] Gudbjartsson D.F., Arnar D.O., Helgadottir A., Gretarsdottir S., Holm H., Sigurdsson A., Jonasdottir A., Baker A., Thorleifsson G., Kristjansson K. (2007). Variants Conferring Risk of Atrial Fibrillation on Chromosome 4q25. Nature.

[41] Chinchilla A., Daimi H., Lozano-Velasco E., Dominguez J.N., Caballero R., Delpón E., Tamargo J., Cinca J., Hove-Madsen L., Aranega A.E. (2011). PITX2 Insufficiency Leads to Atrial Electrical and Structural Remodeling Linked to Arrhythmogenesis. Circ. Cardiovasc. Genet..

[42] Lozano-Velasco E., Hernández-Torres F., Daimi H., Serra S.A., Herraiz A., Hove-Madsen L., Aránega A., Franco D. (2016). Pitx2 Impairs Calcium Handling in a Dose-Dependent Manner by Modulating Wnt Signalling. Cardiovasc. Res..

[43] Gutierrez A., Chung M.K. (2016). Genomics of Atrial Fibrillation. Curr. Cardiol. Rep..

[44] Kirchhof P., Kahr P.C., Kaese S., Piccini I., Vokshi I., Scheld H.-H., Rotering H., Fortmueller L., Laakmann S., Verheule S. (2011). PITX2c Is Expressed in the Adult Left Atrium, and Reducing Pitx2c Expression Promotes Atrial Fibrillation Inducibility and Complex Changes in Gene Expression. Circ. Cardiovasc. Genet..

[45] Taryma-Leśniak O., Bińkowski J., Przybylowicz P.K., Sokolowska K.E., Borowski K., Wojdacz T.K. (2024). Methylation Patterns at the Adjacent CpG Sites within Enhancers Are a Part of Cell Identity. Epigenetics Chromatin.

[46] Stefansson O.A., Sigurpalsdottir B.D., Rognvaldsson S., Halldorsson G.H., Juliusson K., Sveinbjornsson G., Gunnarsson B., Beyter D., Jonsson H., Gudjonsson S.A. (2024). The Correlation between CpG Methylation and Gene Expression Is Driven by Sequence Variants. Nat. Genet..

[47] Ambrosi C., Manzo M., Baubec T. (2017). Dynamics and Context-Dependent Roles of DNA Methylation. J. Mol. Biol..

[48] Mahmood N., Rabbani S.A. (2019). DNA Methylation Readers and Cancer: Mechanistic and Therapeutic Applications. Front. Oncol..

[49] Lin H., Yin X., Xie Z., Lunetta K.L., Lubitz S.A., Larson M.G., Ko D., Magnani J.W., Mendelson M.M., Liu C. (2017). Methylome-Wide Association Study of Atrial Fibrillation in Framingham Heart Study. Sci. Rep..

[50] Felisbino M.B., McKinsey T.A. (2018). Epigenetics in Cardiac Fibrosis: Emphasis on Inflammation and Fibroblast Activation. JACC Basic Transl. Sci..

[51] Bannister A.J., Kouzarides T. (2011). Regulation of Chromatin by Histone Modifications. Cell Res..

[52] López-Hernández L., Toolan-Kerr P., Bannister A.J., Millán-Zambrano G. (2025). Dynamic Histone Modification Patterns Coordinating DNA Processes. Mol. Cell.

[53] Tingare A., Thienpont B., Roderick H.L. (2013). Epigenetics in the Heart: The Role of Histone Modifications in Cardiac Remodelling. Biochem. Soc. Trans..

[54] Lu J., Qian S., Sun Z. (2024). Targeting Histone Deacetylase in Cardiac Diseases. Front. Physiol..

[55] Backs J., Olson E.N. (2006). Control of Cardiac Growth by Histone Acetylation/Deacetylation. Circ. Res..

[56] Nielsen J.B., Thorolfsdottir R.B., Fritsche L.G., Zhou W., Skov M.W., Graham S.E., Herron T.J., McCarthy S., Schmidt E.M., Sveinbjornsson G. (2018). Biobank-Driven Genomic Discovery Yields New Insight into Atrial Fibrillation Biology. Nat. Genet..

[57] Lkhagva B., Chang S.-L., Chen Y.-C., Kao Y.-H., Lin Y.-K., Chiu C.T.-H., Chen S.-A., Chen Y.-J. (2014). Histone Deacetylase Inhibition Reduces Pulmonary Vein Arrhythmogenesis through Calcium Regulation. Int. J. Cardiol..

[58] Victorino J., Alvarez-Franco A., Manzanares M. (2021). Functional Genomics and Epigenomics of Atrial Fibrillation. J. Mol. Cell. Cardiol..

[59] Zhang D., Hu X., Henning R.H., Brundel B.J.J.M. (2016). Keeping up the Balance: Role of HDACs in Cardiac Proteostasis and Therapeutic Implications for Atrial Fibrillation. Cardiovasc. Res..

[60] Jiang X., Liu B., Nie Z., Duan L., Xiong Q., Jin Z., Yang C., Chen Y. (2021). The Role of M6A Modification in the Biological Functions and Diseases. Signal Transduct. Target. Ther..

[61] Shen H., Lan Y., Zhao Y., Shi Y., Jin J., Xie W. (2020). The Emerging Roles of N6-Methyladenosine RNA Methylation in Human Cancers. Biomark. Res..

[62] Chen C., Wang G., Zou Q., Xiong K., Chen Z., Shao B., Liu Y., Xie D., Ji Y. (2025). M(6)A Reader YTHDF2 Governs the Onset of Atrial Fibrillation by Modulating Cacna1c Translation. Sci. China. Life Sci..

[63] Zheng P.-F., Zhou S.-Y., Zhong C.-Q., Zheng Z.-F., Liu Z.-Y., Pan H.-W., Peng J.-Q. (2023). Identification of M6A Regulator-Mediated RNA Methylation Modification Patterns and Key Immune-Related Genes Involved in Atrial Fibrillation. Aging.

[64] Clapier C.R., Iwasa J., Cairns B.R., Peterson C.L. (2017). Mechanisms of Action and Regulation of ATP-Dependent Chromatin-Remodelling Complexes. Nat. Rev. Mol. Cell Biol..

[65] Doñate Puertas R., Arora R., Rome S., Asatryan B., Roderick H.L., Chevalier P. (2021). Epigenetics in Atrial Fibrillation: A Reappraisal. Hear. Rhythm.

[66] Nie Y., Song C., Huang H., Mao S., Ding K., Tang H. (2024). Chromatin Modifiers in Human Disease: From Functional Roles to Regulatory Mechanisms. Mol. Biomed..

[67] McKinsey T.A., Foo R., Anene-Nzelu C.G., Travers J.G., Vagnozzi R.J., Weber N., Thum T. (2023). Emerging Epigenetic Therapies of Cardiac Fibrosis and Remodelling in Heart Failure: From Basic Mechanisms to Early Clinical Development. Cardiovasc. Res..

[68] Kozomara A., Griffiths-Jones S. (2014). MiRBase: Annotating High Confidence MicroRNAs Using Deep Sequencing Data. Nucleic Acids Res..

[69] Ha T.-Y. (2011). MicroRNAs in Human Diseases: From Cancer to Cardiovascular Disease. Immune Netw..

[70] Ardekani A.M., Naeini M.M. (2010). The Role of MicroRNAs in Human Diseases. Avicenna J. Med. Biotechnol..

[71] Chen J.-F., Mandel E.M., Thomson J.M., Wu Q., Callis T.E., Hammond S.M., Conlon F.L., Wang D.-Z. (2006). The Role of MicroRNA-1 and MicroRNA-133 in Skeletal Muscle Proliferation and Differentiation. Nat. Genet..

[72] Jia X., Zheng S., Xie X., Zhang Y., Wang W., Wang Z., Zhang Y., Wang J., Gao M., Hou Y. (2013). MicroRNA-1 Accelerates the Shortening of Atrial Effective Refractory Period by Regulating KCNE1 and KCNB2 Expression: An Atrial Tachypacing Rabbit Model. PLoS ONE.

[73] Girmatsion Z., Biliczki P., Bonauer A., Wimmer-Greinecker G., Scherer M., Moritz A., Bukowska A., Goette A., Nattel S., Hohnloser S.H. (2009). Changes in MicroRNA-1 Expression and IK1 up-Regulation in Human Atrial Fibrillation. Hear. Rhythm.

[74] Li Y.-D., Hong Y.-F., Yusufuaji Y., Tang B.-P., Zhou X.-H., Xu G.-J., Li J.-X., Sun L., Zhang J.-H., Xin Q. (2015). Altered Expression of Hyperpolarization-Activated Cyclic Nucleotide-Gated Channels and MicroRNA-1 and -133 in Patients with Age-Associated Atrial Fibrillation. Mol. Med. Rep..

[75] Lu Y., Hou S., Huang D., Luo X., Zhang J., Chen J., Xu W. (2015). Expression Profile Analysis of Circulating MicroRNAs and Their Effects on Ion Channels in Chinese Atrial Fibrillation Patients. Int. J. Clin. Exp. Med..

[76] Barana A., Matamoros M., Dolz-Gaitón P., Pérez-Hernández M., Amorós I., Núñez M., Sacristán S., Pedraz Á., Pinto Á., Fernández-Avilés F. (2014). Chronic Atrial Fibrillation Increases MicroRNA-21 in Human Atrial Myocytes Decreasing L-Type Calcium Current. Circ. Arrhythm. Electrophysiol..

[77] Lu Y., Zhang Y., Wang N., Pan Z., Gao X., Zhang F., Zhang Y., Shan H., Luo X., Bai Y. (2010). MicroRNA-328 Contributes to Adverse Electrical Remodeling in Atrial Fibrillation. Circulation.

[78] Ling T.-Y., Wang X.-L., Chai Q., Lu T., Stulak J.M., Joyce L.D., Daly R.C., Greason K.L., Wu L.-Q., Shen W.-K. (2017). Regulation of Cardiac CACNB2 by MicroRNA-499: Potential Role in Atrial Fibrillation. BBA Clin..

[79] Chiang D.Y., Kongchan N., Beavers D.L., Alsina K.M., Voigt N., Neilson J.R., Jakob H., Martin J.F., Dobrev D., Wehrens X.H.T. (2014). Loss of MicroRNA-106b-25 Cluster Promotes Atrial Fibrillation by Enhancing Ryanodine Receptor Type-2 Expression and Calcium Release. Circ. Arrhythm. Electrophysiol..

[80] Cañón S., Caballero R., Herraiz-Martínez A., Pérez-Hernández M., López B., Atienza F., Jalife J., Hove-Madsen L., Delpón E., Bernad A. (2016). MiR-208b Upregulation Interferes with Calcium Handling in HL-1 Atrial Myocytes: Implications in Human Chronic Atrial Fibrillation. J. Mol. Cell. Cardiol..

[81] Adam O., Löhfelm B., Thum T., Gupta S.K., Puhl S.-L., Schäfers H.-J., Böhm M., Laufs U. (2012). Role of MiR-21 in the Pathogenesis of Atrial Fibrosis. Basic Res. Cardiol..

[82] Cao W., Shi P., Ge J.-J. (2017). MiR-21 Enhances Cardiac Fibrotic Remodeling and Fibroblast Proliferation via CADM1/STAT3 Pathway. BMC Cardiovasc. Disord..

[83] Dawson K., Wakili R., Ordög B., Clauss S., Chen Y., Iwasaki Y., Voigt N., Qi X.Y., Sinner M.F., Dobrev D. (2013). MicroRNA29: A Mechanistic Contributor and Potential Biomarker in Atrial Fibrillation. Circulation.

[84] Goren Y., Meiri E., Hogan C., Mitchell H., Lebanony D., Salman N., Schliamser J.E., Amir O. (2014). Relation of Reduced Expression of MiR-150 in Platelets to Atrial Fibrillation in Patients with Chronic Systolic Heart Failure. Am. J. Cardiol..

[85] McManus D.D., Tanriverdi K., Lin H., Esa N., Kinno M., Mandapati D., Tam S., Okike O.N., Ellinor P.T., Keaney J.F.J. (2015). Plasma MicroRNAs Are Associated with Atrial Fibrillation and Change after Catheter Ablation (the MiRhythm Study). Hear. Rhythm.

[86] Harling L., Lambert J., Ashrafian H., Darzi A., Gooderham N.J., Athanasiou T. (2017). Elevated Serum MicroRNA 483-5p Levels May Predict Patients at Risk of Post-Operative Atrial Fibrillation. Eur. J. Cardio-Thorac. Surg. Off. J. Eur. Assoc. Cardio-Thorac. Surg..

[87] Shen X.-B., Zhang S.-H., Li H.-Y., Chi X.-D., Jiang L., Huang Q.-L., Xu S.-H. (2018). Rs12976445 Polymorphism Is Associated with Post-Ablation Recurrence of Atrial Fibrillation by Modulating the Expression of MicroRNA-125a and Interleukin-6R. Med. Sci. Monit. Int. Med. J. Exp. Clin. Res..

[88] Wei X.J., Han M., Yang F.Y., Wei G.C., Liang Z.G., Yao H., Ji C.W., Xie R.S., Gong C.L., Tian Y. (2015). Biological Significance of MiR-126 Expression in Atrial Fibrillation and Heart Failure. Braz. J. Med. Biol. Res..

[89] Ren B., Cai S., Wang M. (2025). Risk Factors for Recurrence of Persistent Atrial Fibrillation after Radiofrequency Ablation and Correlation with Plasma MiRNA Expression. Minerva Cardiol. Angiol..

[90] Balan A.I., Halaţiu V.B., Comșulea E., Mutu C.C., Cozac D.A., Aspru I., Păcurar D., Bănescu C., Perian M., Scridon A. (2025). The Diagnostic and Predictive Potential of MiR-328 in Atrial Fibrillation: Insights from a Spontaneously Hypertensive Rat Model. Int. J. Mol. Sci..

[91] Denham N.C., Pearman C.M., Caldwell J.L., Madders G.W.P., Eisner D.A., Trafford A.W., Dibb K.M. (2018). Calcium in the Pathophysiology of Atrial Fibrillation and Heart Failure. Front. Physiol..

[92] Xu J., Cui G., Esmailian F., Plunkett M., Marelli D., Ardehali A., Odim J., Laks H., Sen L. (2004). Atrial Extracellular Matrix Remodeling and the Maintenance of Atrial Fibrillation. Circulation.

[93] Chakraborty P., Farhat K., Po S.S., Armoundas A.A., Stavrakis S. (2023). Autonomic Nervous System and Cardiac Metabolism: Links Between Autonomic and Metabolic Remodeling in Atrial Fibrillation. JACC Clin. Electrophysiol..

[94] Rao M., Hu J., Zhang Y., Gao F., Zhang F., Yang Z., Zhang X., Hou Y. (2018). Time-Dependent Cervical Vagus Nerve Stimulation and Frequency-Dependent Right Atrial Pacing Mediates Induction of Atrial Fibrillation. Anatol. J. Cardiol..

[95] Zhang Y., Zheng S., Geng Y., Xue J., Wang Z., Xie X., Wang J., Zhang S., Hou Y. (2015). MicroRNA Profiling of Atrial Fibrillation in Canines: MiR-206 Modulates Intrinsic Cardiac Autonomic Nerve Remodeling by Regulating SOD1. PLoS ONE.

[96] Srivastava K., Tyagi K. (2018). Single Nucleotide Polymorphisms of MicroRNA in Cardiovascular Diseases. Clin. Chim. Acta.

[97] Króliczewski J., Sobolewska A., Lejnowski D., Collawn J.F., Bartoszewski R. (2018). MicroRNA Single Polynucleotide Polymorphism Influences on MicroRNA Biogenesis and MRNA Target Specificity. Gene.

[98] Su Y.-M., Li J., Guo Y.-F., Cai F., Cai X.-X., Pan H.-Y., Deng X.-T., Pan M. (2015). A Functional Single-Nucleotide Polymorphism in Pre-MicroRNA-196a2 Is Associated with Atrial Fibrillation in Han Chinese. Clin. Lab..

[99] Chodurska B., Kunej T. (2025). Long Non-Coding RNAs in Humans: Classification, Genomic Organization and Function. Non-Coding RNA Res..

[100] Babapoor-Farrokhran S., Gill D., Rasekhi R.T. (2020). The Role of Long Noncoding RNAs in Atrial Fibrillation. Hear. Rhythm.

[101] Dai H., Zhao N., Liu H., Zheng Y., Zhao L. (2021). LncRNA Nuclear-Enriched Abundant Transcript 1 Regulates Atrial Fibrosis via the MiR-320/NPAS2 Axis in Atrial Fibrillation. Front. Pharmacol..

[102] Du J., Li Z., Wang X., Li J., Liu D., Wang X., Wei J., Ma S., Zhang Y., Hou Y. (2020). Long Noncoding RNA TCONS-00106987 Promotes Atrial Electrical Remodelling during Atrial Fibrillation by Sponging MiR-26 to Regulate KCNJ2. J. Cell. Mol. Med..

[103] Li Z., Wang X., Wang W., Du J., Wei J., Zhang Y., Wang J., Hou Y. (2017). Altered Long Non-Coding RNA Expression Profile in Rabbit Atria with Atrial Fibrillation: TCONS_00075467 Modulates Atrial Electrical Remodeling by Sponging MiR-328 to Regulate CACNA1C. J. Mol. Cell. Cardiol..

[104] Ramos K.S., Li J., Wijdeveld L.F.J., van Schie M.S., Taverne Y.J.H.J., Boon R.A., de Groot N.M.S., Brundel B.J.J.M. (2023). Long Noncoding RNA UCA1 Correlates With Electropathology in Patients With Atrial Fibrillation. JACC Clin. Electrophysiol..

[105] Wang H., Song T., Zhao Y., Zhao J., Wang X., Fu X. (2020). Long Non-Coding RNA LICPAR Regulates Atrial Fibrosis via TGF-β/Smad Pathway in Atrial Fibrillation. Tissue Cell.

[106] Ruan Z., Sun X., Sheng H., Zhu L. (2015). Long Non-Coding RNA Expression Profile in Atrial Fibrillation. Int. J. Clin. Exp. Pathol..

[107] Xu Y., Huang R., Gu J., Jiang W. (2016). Identification of Long Non-Coding RNAs as Novel Biomarker and Potential Therapeutic Target for Atrial Fibrillation in Old Adults. Oncotarget.

[108] Mei B., Liu H., Yang S., Liang M.-Y., Yue Y., Huang S.-Q., Hou J., Chen G.-X., Wu Z.-K. (2018). Long Non-Coding RNA Expression Profile in Permanent Atrial Fibrillation Patients with Rheumatic Heart Disease. Eur. Rev. Med. Pharmacol. Sci..

[109] Su Y., Li L., Zhao S., Yue Y., Yang S. (2018). The Long Noncoding RNA Expression Profiles of Paroxysmal Atrial Fibrillation Identified by Microarray Analysis. Gene.

[110] Chen G., Guo H., Song Y., Chang H., Wang S., Zhang M., Liu C. (2016). Long Non-coding RNA AK055347 Is Upregulated in Patients with Atrial Fibrillation and Regulates Mitochondrial Energy Production in Myocardiocytes. Mol. Med. Rep..

[111] Qian C., Li H., Chang D., Wei B., Wang Y. (2019). Identification of Functional LncRNAs in Atrial Fibrillation by Integrative Analysis of the LncRNA-MRNA Network Based on Competing Endogenous RNAs Hypothesis. J. Cell. Physiol..

[112] Zhao L., Ma Z., Guo Z., Zheng M., Li K., Yang X. (2020). Analysis of Long Non-Coding RNA and MRNA Profiles in Epicardial Adipose Tissue of Patients with Atrial Fibrillation. Biomed. Pharmacother..

[113] Lu J., Xu F.-Q., Guo J.-J., Lin P.-L., Meng Z., Hu L.-G., Li J., Li D., Lu X.-H., An Y. (2019). Long Noncoding RNA GAS5 Attenuates Cardiac Fibroblast Proliferation in Atrial Fibrillation via Repressing ALK5. Eur. Rev. Med. Pharmacol. Sci..

[114] Sun F., Guo Z., Zhang C., Che H., Gong W., Shen Z., Shi Y., Ge S. (2019). LncRNA NRON Alleviates Atrial Fibrosis through Suppression of M1 Macrophages Activated by Atrial Myocytes. Biosci. Rep..

[115] Yu X.-J., Zou L.-H., Jin J.-H., Xiao F., Li L., Liu N., Yang J.-F., Zou T. (2017). Long Noncoding RNAs and Novel Inflammatory Genes Determined by RNA Sequencing in Human Lymphocytes Are Up-Regulated in Permanent Atrial Fibrillation. Am. J. Transl. Res..

[116] Shen C., Kong B., Liu Y., Xiong L., Shuai W., Wang G., Quan D., Huang H. (2018). YY1-Induced Upregulation of LncRNA KCNQ1OT1 Regulates Angiotensin II-Induced Atrial Fibrillation by Modulating MiR-384b/CACNA1C Axis. Biochem. Biophys. Res. Commun..

[117] Wen J., Ruan Z.-B., Wang F., Chen G.-C., Zhu J.-G., Ren Y., Zhu L. (2023). Construction of Atrial Fibrillation-Related CircRNA/LncRNA-MiRNA-MRNA Regulatory Network and Analysis of Potential Biomarkers. J. Clin. Lab. Anal..

[118] Feng X.-Y., Zhu S.-X., Pu K.-J., Huang H.-J., Chen Y.-Q., Wang W.-T. (2023). New Insight into CircRNAs: Characterization, Strategies, and Biomedical Applications. Exp. Hematol. Oncol..

[119] Zhou W.-Y., Cai Z.-R., Liu J., Wang D.-S., Ju H.-Q., Xu R.-H. (2020). Circular RNA: Metabolism, Functions and Interactions with Proteins. Mol. Cancer.

[120] Xue Z., Zhu J., Liu J., Wang L., Ding J. (2023). Circular RNAs in Atrial Fibrillation: From Bioinformatics Analysis of CircRNA-MiRNA-MRNA Network to Serum Expression. Biochem. Biophys. Rep..

[121] Liu S.-S., Guo H.-Y., Zhu J., Ma J.-L., Liu S.-Z., He K.-L., Bian S.-Y. (2023). Circulating CircRNA Expression Profile and Its Potential Role in Late Recurrence of Paroxysmal Atrial Fibrillation Post Catheter Ablation. J. Geriatr. Cardiol..

[122] Hu M., Wei X., Li M., Tao L., Wei L., Zhang M., Cheng H., Yuan Y. (2019). Circular RNA Expression Profiles of Persistent Atrial Fibrillation in Patients with Rheumatic Heart Disease. Anatol. J. Cardiol..

[123] Costa M.C., Cortez-Dias N., Gabriel A., de Sousa J., Fiúza M., Gallego J., Nobre Â., Pinto F.J., Enguita F.J. (2019). CircRNA-MiRNA Cross-Talk in the Transition from Paroxysmal to Permanent Atrial Fibrillation. Int. J. Cardiol..

[124] Ruan Z.-B., Wang F., Bao T.-T., Yu Q.-P., Chen G.-C., Zhu L. (2020). Genome-Wide Analysis of Circular RNA Expression Profiles in Patients with Atrial Fibrillation. Int. J. Clin. Exp. Pathol..

[125] Zhu X., Tang X., Chong H., Cao H., Fan F., Pan J., Wang D., Zhou Q. (2020). Expression Profiles of Circular RNA in Human Atrial Fibrillation With Valvular Heart Diseases. Front. Cardiovasc. Med..

[126] Du W.W., Rafiq M., Yuan H., Li X., Wang S., Wu J., Wei J., Li R.-K., Guo H., Yang B.B. (2025). A Novel Protein NAB1-356 Encoded by CircRNA CircNAB1 Mitigates Atrial Fibrillation by Reducing Inflammation and Fibrosis. Adv. Sci..

[127] Shangguan W., Liang X., Shi W., Liu T., Wang M., Li G. (2018). Identification and Characterization of Circular RNAs in Rapid Atrial Pacing Dog Atrial Tissue. Biochem. Biophys. Res. Commun..

[128] Zhang Y., Shen H., Wang P., Min J., Yu Y., Wang Q., Wang S., Xi W., Nguyen Q.M., Xiao J. (2020). Identification and Characterization of Circular RNAs in Atrial Appendage of Patients with Atrial Fibrillation. Exp. Cell Res..

[129] Jiang S., Guo C., Zhang W., Che W., Zhang J., Zhuang S., Wang Y., Zhang Y., Liu B. (2019). The Integrative Regulatory Network of CircRNA, MicroRNA, and MRNA in Atrial Fibrillation. Front. Genet..

[130] Liu T., Zhang G., Wang Y., Rao M., Zhang Y., Guo A., Wang M. (2020). Identification of Circular RNA-MicroRNA-Messenger RNA Regulatory Network in Atrial Fibrillation by Integrated Analysis. Biomed Res. Int..

[131] Wang Y., Liu B. (2020). Circular RNA in Diseased Heart. Cells.

[132] Zhang P.-P., Sun J., Li W. (2020). Genome-Wide Profiling Reveals Atrial Fibrillation-Related Circular RNAs in Atrial Appendages. Gene.

[133] Hu X., Chen L., Wu S., Xu K., Jiang W., Qin M., Zhang Y., Liu X. (2019). Integrative Analysis Reveals Key Circular RNA in Atrial Fibrillation. Front. Genet..

[134] Lozano-Velasco E., Franco D., Aranega A., Daimi H. (2020). Genetics and Epigenetics of Atrial Fibrillation. Int. J. Mol. Sci..

[135] Li D., Nie J., Han Y., Ni L. (2021). Epigenetic Mechanism and Therapeutic Implications of Atrial Fibrillation. Front. Cardiovasc. Med..

[136] Dai D.-F., Rabinovitch P.S. (2009). Cardiac Aging in Mice and Humans: The Role of Mitochondrial Oxidative Stress. Trends Cardiovasc. Med..

[137] Korantzopoulos P., Kolettis T.M., Galaris D., Goudevenos J.A. (2007). The Role of Oxidative Stress in the Pathogenesis and Perpetuation of Atrial Fibrillation. Int. J. Cardiol..

[138] Lv L., Chen Q., Lu J., Zhao Q., Wang H., Li J., Yuan K., Dong Z. (2024). Potential Regulatory Role of Epigenetic Modifications in Aging-Related Heart Failure. Int. J. Cardiol..

[139] Grzeczka A., Graczyk S., Kordowitzki P. (2023). DNA Methylation and Telomeres-Their Impact on the Occurrence of Atrial Fibrillation during Cardiac Aging. Int. J. Mol. Sci..

[140] Roberts J.D., Vittinghoff E., Lu A.T., Alonso A., Wang B., Sitlani C.M., Mohammadi-Shemirani P., Fornage M., Kornej J., Brody J.A. (2021). Epigenetic Age and the Risk of Incident Atrial Fibrillation. Circulation.

[141] Doñate Puertas R., Meugnier E., Romestaing C., Rey C., Morel E., Lachuer J., Gadot N., Scridon A., Julien C., Tronc F. (2017). Atrial Fibrillation Is Associated with Hypermethylation in Human Left Atrium, and Treatment with Decitabine Reduces Atrial Tachyarrhythmias in Spontaneously Hypertensive Rats. Transl. Res..

[142] Fatima N., Schooley J.F.J., Claycomb W.C., Flagg T.P. (2012). Promoter DNA Methylation Regulates Murine SUR1 (Abcc8) and SUR2 (Abcc9) Expression in HL-1 Cardiomyocytes. PLoS ONE.

[143] Shen K., Tu T., Yuan Z., Yi J., Zhou Y., Liao X., Liu Q., Zhou X. (2017). DNA Methylation Dysregulations in Valvular Atrial Fibrillation. Clin. Cardiol..

[144] Zhang Y., Ren J. (2016). Epigenetics and Obesity Cardiomyopathy: From Pathophysiology to Prevention and Management. Pharmacol. Ther..

[145] Takahashi K., Sasano T., Sugiyama K., Kurokawa J., Tamura N., Soejima Y., Sawabe M., Isobe M., Furukawa T. (2016). High-Fat Diet Increases Vulnerability to Atrial Arrhythmia by Conduction Disturbance via MiR-27b. J. Mol. Cell. Cardiol..

[146] Guedes E.C., França G.S., Lino C.A., Koyama F.C., Moreira L.D., Alexandre J.G., Barreto-Chaves M.L.M., Galante P.A.F., Diniz G.P. (2016). MicroRNA Expression Signature Is Altered in the Cardiac Remodeling Induced by High Fat Diets. J. Cell. Physiol..

[147] Nalliah C.J., Sanders P., Kottkamp H., Kalman J.M. (2016). The Role of Obesity in Atrial Fibrillation. Eur. Heart J..

[148] Kantharia B.K., Tabary M., Wu L., Wang X., Narasimhan B., Linz D., Heijman J., Wehrens X.H.T. (2025). Diabetes and Atrial Fibrillation: Insight From Basic to Translational Science Into the Mechanisms and Management. J. Cardiovasc. Electrophysiol..

[149] Ma X., Mei S., Wuyun Q., Zhou L., Sun D., Yan J. (2024). Epigenetics in Diabetic Cardiomyopathy. Clin. Epigenetics.

[150] Li Y., Liu Y., Liu S., Gao M., Wang W., Chen K., Huang L., Liu Y. (2023). Diabetic Vascular Diseases: Molecular Mechanisms and Therapeutic Strategies. Signal Transduct. Target. Ther..

[151] Chavali V., Tyagi S.C., Mishra P.K. (2012). MicroRNA-133a Regulates DNA Methylation in Diabetic Cardiomyocytes. Biochem. Biophys. Res. Commun..

[152] Xu X., Tan X., Tampe B., Nyamsuren G., Liu X., Maier L.S., Sossalla S., Kalluri R., Zeisberg M., Hasenfuss G. (2015). Epigenetic Balance of Aberrant Rasal1 Promoter Methylation and Hydroxymethylation Regulates Cardiac Fibrosis. Cardiovasc. Res..

[153] Pan J.-A., Lin H., Yu J.-Y., Zhang H.-L., Zhang J.-F., Wang C.-Q., Gu J. (2021). MiR-21-3p Inhibits Adipose Browning by Targeting FGFR1 and Aggravates Atrial Fibrosis in Diabetes. Oxid. Med. Cell. Longev..

[154] Kantharia B.K., Zhao S., Linz D., Heijman J., Wehrens X.H.T. (2025). Hypertension and Atrial Fibrillation: Insight From Basic to Translational Science Into the Mechanisms and Management. J. Cardiovasc. Electrophysiol..

[155] Antoun I., Layton G.R., Nizam A., Barker J., Abdelrazik A., Eldesouky M., Koya A., Lau E.Y.M., Zakkar M., Somani R. (2025). Hypertension and Atrial Fibrillation: Bridging the Gap Between Mechanisms, Risk, and Therapy. Medicina.

[156] Shi Y., Zhang H., Huang S., Yin L., Wang F., Luo P., Huang H. (2022). Epigenetic Regulation in Cardiovascular Disease: Mechanisms and Advances in Clinical Trials. Signal Transduct. Target. Ther..

[157] Kao Y.-H., Chen Y.-C., Chung C.-C., Lien G.-S., Chen S.-A., Kuo C.-C., Chen Y.-J. (2013). Heart Failure and Angiotensin II Modulate Atrial Pitx2c Promotor Methylation. Clin. Exp. Pharmacol. Physiol..

[158] Shafi O., Zahra K., Shah H.H. (2024). Dysregulations in Cardiogenic Mechanisms by TGF-Beta and Angiotensin II in Cardiac Remodeling Post-Ischemic Injury: A Systematic Review. medRxiv.

[159] Yuntao F., Jinjun L., Hua Fen L., Huiyu C., Dishiwen L., Zhen C., Wang Y., Wang X., Ke Y., Yanni C. (2024). Atrial Fibroblast-Derived Exosomal MiR-21 Upregulate Myocardial KCa3.1 via the PI3K-Akt Pathway during Rapid Pacing. Heliyon.

[160] Watanabe K., Narumi T., Watanabe T., Otaki Y., Takahashi T., Aono T., Goto J., Toshima T., Sugai T., Wanezaki M. (2020). The Association between MicroRNA-21 and Hypertension-Induced Cardiac Remodeling. PLoS ONE.

[161] van Rooij E., Sutherland L.B., Qi X., Richardson J.A., Hill J., Olson E.N. (2007). Control of Stress-Dependent Cardiac Growth and Gene Expression by a MicroRNA. Science.

[162] Montgomery R.L., Hullinger T.G., Semus H.M., Dickinson B.A., Seto A.G., Lynch J.M., Stack C., Latimer P.A., Olson E.N., van Rooij E. (2011). Therapeutic Inhibition of MiR-208a Improves Cardiac Function and Survival during Heart Failure. Circulation.

[163] Park H. (2017). Hypoxia Suffocates Histone Demethylases to Change Gene Expression: A Metabolic Control of Histone Methylation. BMB Rep..

[164] Zhao Y., Xiong W., Li C., Zhao R., Lu H., Song S., Zhou Y., Hu Y., Shi B., Ge J. (2023). Hypoxia-Induced Signaling in the Cardiovascular System: Pathogenesis and Therapeutic Targets. Signal Transduct. Target. Ther..

[165] Zhou F., Zhou J.-B., Wei T.-P., Wu D., Wang R.-X. (2025). The Role of HIF-1α in Atrial Fibrillation: Recent Advances and Therapeutic Potentials. Rev. Cardiovasc. Med..

[166] Watson C.J., Collier P., Tea I., Neary R., Watson J.A., Robinson C., Phelan D., Ledwidge M.T., McDonald K.M., McCann A. (2014). Hypoxia-Induced Epigenetic Modifications Are Associated with Cardiac Tissue Fibrosis and the Development of a Myofibroblast-like Phenotype. Hum. Mol. Genet..

[167] Grimaldi V., De Pascale M.R., Zullo A., Soricelli A., Infante T., Mancini F.P., Napoli C. (2017). Evidence of Epigenetic Tags in Cardiac Fibrosis. J. Cardiol..

[168] Shao J., Liu J., Zuo S. (2022). Roles of Epigenetics in Cardiac Fibroblast Activation and Fibrosis. Cells.

[169] Hancock R.L., Dunne K., Walport L.J., Flashman E., Kawamura A. (2015). Epigenetic Regulation by Histone Demethylases in Hypoxia. Epigenomics.

[170] Varrias D., Kossack A., Leavitt J., Chhetri C., Roselli V., Velichkovikj S., Altschul E., Bhasin K., Mina B., Oks M. (2025). Adherence to CPAP for Patients with Atrial Fibrillation Undergoing Catheter Ablation: A “Real-World” Analysis. Hear. Rhythm.

[171] Li F., He C.-J., Ding C.-H., Wang R.-X., Li H. (2023). Continuous Positive Airway Pressure Therapy Might Be an Effective Strategy on Reduction of Atrial Fibrillation Recurrence after Ablation in Patients with Obstructive Sleep Apnea: Insights from the Pooled Studies. Front. Neurol..

[172] Lee J.-W., Roh S.-Y., Yoon W.-S., Kim J., Jo E., Bae D.-H., Kim M., Lee J.-H., Kim S.M., Choi W.G. (2024). Changes in Alcohol Consumption Habits and Risk of Atrial Fibrillation: A Nationwide Population-Based Study. Eur. J. Prev. Cardiol..

[173] Natarajan S.K., Pachunka J.M., Mott J.L. (2015). Role of MicroRNAs in Alcohol-Induced Multi-Organ Injury. Biomolecules.

[174] Yeligar S., Tsukamoto H., Kalra V.K. (2009). Ethanol-Induced Expression of ET-1 and ET-BR in Liver Sinusoidal Endothelial Cells and Human Endothelial Cells Involves Hypoxia-Inducible Factor-1alpha and MicrorNA-199. J. Immunol..

[175] Wan Y., Slevin E., Koyama S., Huang C.-K., Shetty A.K., Li X., Harrison K., Li T., Zhou B., Lorenzo S.R. (2023). MiR-34a Regulates Macrophage-Associated Inflammation and Angiogenesis in Alcohol-Induced Liver Injury. Hepatol. Commun..

[176] Yamakuchi M., Ferlito M., Lowenstein C.J. (2008). MiR-34a Repression of SIRT1 Regulates Apoptosis. Proc. Natl. Acad. Sci. USA.

[177] Takahashi Y., Nitta J., Kobori A., Sakamoto Y., Nagata Y., Tanimoto K., Matsuo S., Yamane T., Morita N., Satomi K. (2021). Alcohol Consumption Reduction and Clinical Outcomes of Catheter Ablation for Atrial Fibrillation. Circ. Arrhythm. Electrophysiol..

[178] Xie M., Hill J.A. (2013). HDAC-Dependent Ventricular Remodeling. Trends Cardiovasc. Med..

[179] Kee H.J., Sohn I.S., Nam K.I., Park J.E., Qian Y.R., Yin Z., Ahn Y., Jeong M.H., Bang Y.-J., Kim N. (2006). Inhibition of Histone Deacetylation Blocks Cardiac Hypertrophy Induced by Angiotensin II Infusion and Aortic Banding. Circulation.

[180] Zhang D., Wu C.-T., Qi X., Meijering R.A.M., Hoogstra-Berends F., Tadevosyan A., Cubukcuoglu Deniz G., Durdu S., Akar A.R., Sibon O.C.M. (2014). Activation of Histone Deacetylase-6 Induces Contractile Dysfunction through Derailment of α-Tubulin Proteostasis in Experimental and Human Atrial Fibrillation. Circulation.

[181] Huang K., Zhang Q., Ruan H., Guo C., Wu S., Liu Q., Zhang D., Long S., Wang W., Wu Z. (2024). Pazopanib Attenuated Bleomycin-Induced Pulmonary Fibrosis via Suppressing TGF-Β1 Signaling Pathway. J. Thorac. Dis..

[182] Peng D., Fu M., Wang M., Wei Y., Wei X. (2022). Targeting TGF-β Signal Transduction for Fibrosis and Cancer Therapy. Mol. Cancer.

[183] Tao H., Yang J.-J., Hu W., Shi K.-H., Li J. (2016). HDAC6 Promotes Cardiac Fibrosis Progression through Suppressing RASSF1A Expression. Cardiology.

[184] McKinsey T.A. (2011). Isoform-Selective HDAC Inhibitors: Closing in on Translational Medicine for the Heart. J. Mol. Cell. Cardiol..

[185] Williams S.M., Golden-Mason L., Ferguson B.S., Schuetze K.B., Cavasin M.A., Demos-Davies K., Yeager M.E., Stenmark K.R., McKinsey T.A. (2014). Class I HDACs Regulate Angiotensin II-Dependent Cardiac Fibrosis via Fibroblasts and Circulating Fibrocytes. J. Mol. Cell. Cardiol..

[186] Fang J., Shu S., Dong H., Yue X., Piao J., Li S., Hong L., Cheng X.W. (2024). Histone Deacetylase 6 Controls Cardiac Fibrosis and Remodelling through the Modulation of TGF-Β1/Smad2/3 Signalling in Post-Infarction Mice. J. Cell. Mol. Med..

[187] Vardas E.P., Theofilis P., Oikonomou E., Vardas P.E., Tousoulis D. (2024). MicroRNAs in Atrial Fibrillation: Mechanisms, Vascular Implications, and Therapeutic Potential. Biomedicines.

[188] van den Berg N.W.E., Kawasaki M., Berger W.R., Neefs J., Meulendijks E., Tijsen A.J., de Groot J.R. (2017). MicroRNAs in Atrial Fibrillation: From Expression Signatures to Functional Implications. Cardiovasc. Drugs Ther..

[189] Garreau M., Weidner J., Hamilton R., Kolosionek E., Toki N., Stavenhagen K., Paris C., Bonetti A., Czechtizky W., Gnerlich F. (2024). Chemical Modification Patterns for MicroRNA Therapeutic Mimics: A Structure-Activity Relationship (SAR) Case-Study on MiR-200c. Nucleic Acids Res..

[190] Khalaji A., Mehrtabar S., Jabraeilipour A., Doustar N., Rahmani Youshanlouei H., Tahavvori A., Fattahi P., Alavi S.M.A., Taha S.R., Fazlollahpour-Naghibi A. (2024). Inhibitory Effect of MicroRNA-21 on Pathways and Mechanisms Involved in Cardiac Fibrosis Development. Ther. Adv. Cardiovasc. Dis..

[191] Raucci A., Macrì F., Castiglione S., Badi I., Vinci M.C., Zuccolo E. (2021). MicroRNA-34a: The Bad Guy in Age-Related Vascular Diseases. Cell. Mol. Life Sci..

[192] Chioccioli M., Roy S., Newell R., Pestano L., Dickinson B., Rigby K., Herazo-Maya J., Jenkins G., Ian S., Saini G. (2022). A Lung Targeted MiR-29 Mimic as a Therapy for Pulmonary Fibrosis. EBioMedicine.

[193] Fan X., Gao Y., Zhang X., Lughmani H.Y., Kennedy D.J., Haller S.T., Pierre S.V., Shapiro J.I., Tian J. (2020). A Strategic Expression Method of MiR-29b and Its Anti-Fibrotic Effect Based on RNA-Sequencing Analysis. PLoS ONE.

[194] Ahmadi S.E., Soleymani M., Shahriyary F., Amirzargar M.R., Ofoghi M., Fattahi M.D., Safa M. (2023). Viral Vectors and Extracellular Vesicles: Innate Delivery Systems Utilized in CRISPR/Cas-Mediated Cancer Therapy. Cancer Gene Ther..

[195] Iqbal Z., Rehman K., Mahmood A., Shabbir M., Liang Y., Duan L., Zeng H. (2024). Exosome for MRNA Delivery: Strategies and Therapeutic Applications. J. Nanobiotechnol..

[196] Kang J.-Y., Park H., Kim H., Mun D., Park H., Yun N., Joung B. (2019). Human Peripheral Blood-derived Exosomes for MicroRNA Delivery. Int. J. Mol. Med..

[197] Policarpi C., Munafò M., Tsagkris S., Carlini V., Hackett J.A. (2024). Systematic Epigenome Editing Captures the Context-Dependent Instructive Function of Chromatin Modifications. Nat. Genet..

[198] Nishiga M., Liu C., Qi L.S., Wu J.C. (2022). The Use of New CRISPR Tools in Cardiovascular Research and Medicine. Nat. Rev. Cardiol..

[199] Qian J., Liu S.X. (2024). CRISPR/DCas9-Tet1-Mediated DNA Methylation Editing. Bio-Protocol.

[200] Cai R., Lv R., Shi X., Yang G., Jin J. (2023). CRISPR/DCas9 Tools: Epigenetic Mechanism and Application in Gene Transcriptional Regulation. Int. J. Mol. Sci..

[201] Kang J.G., Park J.S., Ko J.-H., Kim Y.-S. (2019). Regulation of Gene Expression by Altered Promoter Methylation Using a CRISPR/Cas9-Mediated Epigenetic Editing System. Sci. Rep..

[202] Tarifa C., Serra S.A., Herraiz-Martínez A., Lozano-Velasco E., Benítez R., Aranega A., Franco D., Hove-Madsen L. (2023). Pitx2c Deficiency Confers Cellular Electrophysiological Hallmarks of Atrial Fibrillation to Isolated Atrial Myocytes. Biomed. Pharmacother..

[203] Kim K., Blackwell D.J., Yuen S.L., Thorpe M.P., Johnston J.N., Cornea R.L., Knollmann B.C. (2023). The Selective RyR_2_ Inhibitor Ent-Verticilide Suppresses Atrial Fibrillation Susceptibility Caused by Pitx_2_ Deficiency. J. Mol. Cell. Cardiol..

[204] Schulz C., Lemoine M.D., Mearini G., Koivumäki J., Sani J., Schwedhelm E., Kirchhof P., Ghalawinji A., Stoll M., Hansen A. (2023). PITX2 Knockout Induces Key Findings of Electrical Remodeling as Seen in Persistent Atrial Fibrillation. Circ. Arrhythm. Electrophysiol..

[205] Li T., Yang Y., Qi H., Cui W., Zhang L., Fu X., He X., Liu M., Li P.-F., Yu T. (2023). CRISPR/Cas9 Therapeutics: Progress and Prospects. Signal Transduct. Target. Ther..

[206] Karakasis P., Theofilis P., Patoulias D., Vlachakis P.K., Pamporis K., Sagris M., Ktenopoulos N., Kassimis G., Antoniadis A.P., Fragakis N. (2025). Sodium–Glucose Cotransporter 2 Inhibitors in Aortic Stenosis: Toward a Comprehensive Cardiometabolic Approach. Int. J. Mol. Sci..

[207] Mylonas N., Nikolaou P.E., Karakasis P., Stachteas P., Fragakis N., Andreadou I. (2024). Endothelial Protection by Sodium-Glucose Cotransporter 2 Inhibitors: A Literature Review of In Vitro and In Vivo Studies. Int. J. Mol. Sci..

[208] Karakasis P., Pamporis K., Stachteas P., Patoulias D., Bougioukas K.I., Fragakis N. (2023). Efficacy and Safety of Sodium-Glucose Cotransporter-2 Inhibitors in Heart Failure with Mildly Reduced or Preserved Ejection Fraction: An Overview of 36 Systematic Reviews. Heart Fail. Rev..

[209] Stachteas P., Nasoufidou A., Patoulias D., Karakasis P., Karagiannidis E., Mourtzos M.-A., Samaras A., Apostolidou X., Fragakis N. (2024). The Role of Sodium-Glucose Co-Transporter-2 Inhibitors on Diuretic Resistance in Heart Failure. Int. J. Mol. Sci..

[210] Stachteas P., Nasoufidou A., Karagiannidis E., Patoulias D., Karakasis P., Alexiou S., Samaras A., Zormpas G., Stavropoulos G., Tsalikakis D. (2024). The Role of Sodium Glucose Co-Transporter 2 Inhibitors in Atrial Fibrillation: A Comprehensive Review. J. Clin. Med..

[211] Karakasis P., Theofilis P., Patoulias D., Schuermans A., Vlachakis P.K., Klisic A., Rizzo M., Fragakis N. (2025). Sodium-Glucose Cotransporter 2 Inhibitors and Outcomes in Transthyretin Amyloid Cardiomyopathy: Systematic Review and Meta-Analysis. Eur. J. Clin. Investig..

[212] Theofilis P., Oikonomou E., K Vlachakis P., Karakasis P., Dimitriadis K., Sagris M., Pamporis K., Drakopoulou M., Siasos G., Tsioufis K. (2025). Sodium-Glucose Cotransporter 2 Inhibitors and Changes in Epicardial Adipose Tissue: A Systematic Literature Review And Meta-Analysis. Curr. Vasc. Pharmacol..

[213] Karakasis P., Patoulias D., Kassimis G., Koufakis T., Klisic A., Doumas M., Fragakis N., Rizzo M. (2024). Therapeutic Potential of Sodium-Glucose Co-Transporter-2 Inhibitors and Glucagon-like Peptide-1 Receptor Agonists for Patients with Acute Coronary Syndrome: A Review of Clinical Evidence. Curr. Pharm. Des..

[214] Marques F.Z., Vizi D., Khammy O., Mariani J.A., Kaye D.M. (2016). The Transcardiac Gradient of Cardio-MicroRNAs in the Failing Heart. Eur. J. Heart Fail..

[215] Mone P., Lombardi A., Kansakar U., Varzideh F., Jankauskas S.S., Pansini A., Marzocco S., De Gennaro S., Famiglietti M., Macina G. (2023). Empagliflozin Improves the MicroRNA Signature of Endothelial Dysfunction in Patients with Heart Failure with Preserved Ejection Fraction and Diabetes. J. Pharmacol. Exp. Ther..

[216] Lai L., Chen J., Wang N., Zhu G., Duan X., Ling F. (2017). MiRNA-30e Mediated Cardioprotection of ACE2 in Rats with Doxorubicin-Induced Heart Failure through Inhibiting Cardiomyocytes Autophagy. Life Sci..

[217] Solini A., Seghieri M., Giannini L., Biancalana E., Parolini F., Rossi C., Dardano A., Taddei S., Ghiadoni L., Bruno R.M. (2019). The Effects of Dapagliflozin on Systemic and Renal Vascular Function Display an Epigenetic Signature. J. Clin. Endocrinol. Metab..

[218] el Azzouzi H., Leptidis S., Dirkx E., Hoeks J., van Bree B., Brand K., McClellan E.A., Poels E., Sluimer J.C., van den Hoogenhof M.M.G. (2013). The Hypoxia-Inducible MicroRNA Cluster MiR-199a∼214 Targets Myocardial PPARδ and Impairs Mitochondrial Fatty Acid Oxidation. Cell Metab..

[219] Nishitani S., Fukuhara A., Shin J., Okuno Y., Otsuki M., Shimomura I. (2018). Metabolomic and Microarray Analyses of Adipose Tissue of Dapagliflozin-Treated Mice, and Effects of 3-Hydroxybutyrate on Induction of Adiponectin in Adipocytes. Sci. Rep..

[220] Martinez-Moreno J.M., Fontecha-Barriuso M., Martin-Sanchez D., Guerrero-Mauvecin J., Goma-Garces E., Fernandez-Fernandez B., Carriazo S., Sanchez-Niño M.D., Ramos A.M., Ruiz-Ortega M. (2020). Epigenetic Modifiers as Potential Therapeutic Targets in Diabetic Kidney Disease. Int. J. Mol. Sci..

[221] Morciano C., Gugliandolo S., Capece U., Di Giuseppe G., Mezza T., Ciccarelli G., Soldovieri L., Brunetti M., Avolio A., Splendore A. (2024). SGLT2 Inhibition and Adipose Tissue Metabolism: Current Outlook and Perspectives. Cardiovasc. Diabetol..

